# Stochastic Dispersal Rather Than Deterministic Selection Explains the Spatio-Temporal Distribution of Soil Bacteria in a Temperate Grassland

**DOI:** 10.3389/fmicb.2020.01391

**Published:** 2020-06-30

**Authors:** Tim Richter-Heitmann, Benjamin Hofner, Franz-Sebastian Krah, Johannes Sikorski, Pia K. Wüst, Boyke Bunk, Sixing Huang, Kathleen M. Regan, Doreen Berner, Runa S. Boeddinghaus, Sven Marhan, Daniel Prati, Ellen Kandeler, Jörg Overmann, Michael W. Friedrich

**Affiliations:** ^1^Microbial Ecophysiology Group, Faculty of Biology/Chemistry, University of Bremen, Bremen, Germany; ^2^International Max Planck Research School of Marine Microbiology, Max Planck Institute for Marine Microbiology, Bremen, Germany; ^3^Institut für Medizininformatik, Biometrie und Epidemiologie, Friedrich-Alexander-Universität Erlangen-Nürnberg, Erlangen, Germany; ^4^Biodiversity Conservation, Institute for Ecology, Evolution and Diversity, Biologicum, Goethe University Frankfurt, Frankfurt am Main, Germany; ^5^Leibniz Institute DSMZ-German Collection of Microorganisms and Cell Cultures, Braunschweig, Germany; ^6^Institute of Soil Science and Land Evaluation, Soil Biology Department, University of Hohenheim, Stuttgart, Germany; ^7^Institute of Plant Sciences, University of Bern, Bern, Switzerland

**Keywords:** spatio-temporal analysis, soil bacteria communities, community assembly, variable selection, generalized additive model

## Abstract

Spatial and temporal processes shaping microbial communities are inseparably linked but rarely studied together. By Illumina 16S rRNA sequencing, we monitored soil bacteria in 360 stations on a 100 square meter plot distributed across six intra-annual samplings in a rarely managed, temperate grassland. Using a multi-tiered approach, we tested the extent to which stochastic or deterministic processes influenced the composition of local communities. A combination of phylogenetic turnover analysis and null modeling demonstrated that either homogenization by unlimited stochastic dispersal or scenarios, in which neither stochastic processes nor deterministic forces dominated, explained local assembly processes. Thus, the majority of all sampled communities (82%) was rather homogeneous with no significant changes in abundance-weighted composition. However, we detected strong and uniform taxonomic shifts within just nine samples in early summer. Thus, community snapshots sampled from single points in time or space do not necessarily reflect a representative community state. The potential for change despite the overall homogeneity was further demonstrated when the focus shifted to the rare biosphere. Rare OTU turnover, rather than nestedness, characterized abundance-independent β-diversity. Accordingly, boosted generalized additive models encompassing spatial, temporal and environmental variables revealed strong and highly diverse effects of space on OTU abundance, even within the same genus. This pure spatial effect increased with decreasing OTU abundance and frequency, whereas soil moisture – the most important environmental variable – had an opposite effect by impacting abundant OTUs more than the rare ones. These results indicate that – despite considerable oscillation in space and time – the abundant and resident OTUs provide a community backbone that supports much higher β-diversity of a dynamic rare biosphere. Our findings reveal complex interactions among space, time, and environmental filters within bacterial communities in a long-established temperate grassland.

## Introduction

Microbial communities change with increasing spatial distance, as demonstrated for many microbial taxa (Martiny et al., 2006; Ramette and Tiedje, 2007); from the micro- (Raynaud and Nunan, 2014) to landscape- and continental scales (Martiny et al., 2011; Karimi et al., 2018). Likewise, temporal variability is a regular feature of microbial communities, and, for instance, is associated with seasonality (Buckley and Schmidt, 2003; Docherty et al., 2015; Zifcakova et al., 2016) and is a characteristic property of the rare biosphere (Shade et al., 2014; Shade and Gilbert, 2015). The extent to which these changes in community structure and composition are driven by deterministic (i.e., species filtering by environmental adaptation and inter-species interactions) or stochastic processes (i.e., trait- and selection-independent community assembly regulated by stochastic events, such as random proliferation, death, and dispersal) (Stegen et al., 2013; Zhou and Ning, 2017) remains unclear. Both types of processes interact in the assembly of microbial communities (Chase, 2014), but it appears that, in general, environmental filtering dominates (Hanson et al., 2012; Wang et al., 2013; Bahram et al., 2018). Nevertheless, stochastic assembly principles are promoted under certain environmental (Bahram et al., 2016; Wang et al., 2017; Tripathi et al., 2018) and biotic conditions, e.g., increasing body size (Zinger et al., 2018) or community cohesion (Danczak et al., 2018). The ratio between stochastic and deterministic effects depends on the studied taxa (Székely and Langenheder, 2014; Powell et al., 2015; Andam et al., 2016), their abundance and relative frequency (Mo et al., 2018), the spatial scales at which they are studied (Shi et al., 2018), and the stage of microbial community assembly (Langenheder and Székely, 2011; Ferrenberg et al., 2013; Dini-Andreote et al., 2015; Veach et al., 2016). In other words, recent community assembly studies report highly inconsistent results (Langenheder and Lindström, 2019), possibly because they cover a wide array of habitat types and experimental designs. However, disentangling deterministic and stochastic community assembly *in situ* has major implications: The interactions between these two process types impact the biogeochemical functionality of microbial communities (Pholchan et al., 2013; Graham and Stegen, 2017; Zhang et al., 2019), e.g., in the case of strong dispersal of maladapted species overriding local environmental selection.

The formation of spatially heterogeneous species distributions obviously depends on time, as the pace and type of the community assembly processes are likely not constant [e.g., past stochastic events, historical dispersal limitations, or fluxes in environmental heterogeneity (Martiny, 2016)]. Consequently, spatio-temporal sampling designs should be a major focus of environmental microbiome studies. However, many experiments that study spatial distributions of microbes do so at only a single time point, leaving uncertainty about whether the observed communities are in a temporally robust state, or whether seasonal effects have changed the community before or will change it thereafter. It is equally difficult to evaluate the ecological importance of temporal community shifts without knowing the spatial scale at which they operate. There is a rich body of studies covering either spatial or temporal variability of soil microbiomes, but investigations of both dimensions simultaneously are rare, especially at the plot scale. Studies have often explicitly selected sites with strong habitat and/or seasonal turnover on small scales and found effects correlating with this high environmental variability present in their sites (Mummey and Stahl, 2003; Henneberger et al., 2015; Hill et al., 2015; Kivlin and Hawkes, 2016; Albright et al., 2019b). Importantly, none of these study designs were used to study assembly processes of bacteria. Thus, several questions regarding the spatio-temporal variability of microbial soil communities at medium scales (e.g., meter and months) remain unanswered, especially in scenarios with less obvious habitat turnover. How temporally uniform are spatial distributions of microorganisms across time and how constant are those over several meters if the physical macro-environment does not change? Is it possible to monitor the interactions between stochastic and deterministic processes in these cases?

To address these questions, the SCALEMIC experiment was established (Regan et al., 2014), in which the top soil of a temperate grassland was repeatedly sampled on a dense grid within a single year. In the present paper, we used this sampling experiment to disentangle assembly processes of soil bacterial communities, using 16S rRNA as a phylogenetic marker of transcriptional activity potential, which in many bacteria can also correlate with cell growth (Kerkhof and Kemp, 1999; Worden and Binder, 2003). Despite its limitations (Blazewicz et al., 2013), rRNA is still the only marker that provides phylogenetic resolution while enabling the detection of potentially active bacteria, i.e., those being capable of protein biosynthesis, either in resting or metabolically active, but in any case viable, cell states. This is difficult to achieve with DNA, as the presence of relic DNA may obscure patterns of spatial or – particularly – temporal variability in soil microbiomes (Blagodatskaya and Kuzyakov, 2013; Carini et al., 2016; Fierer, 2017). The impact of important environmental variables on microbial community composition and the contribution of rare microbes to ecosystem functionality may be underestimated when studying rRNA genes only (Campbell et al., 2011; Baldrian et al., 2012; Barnard et al., 2015; Jousset et al., 2017). Since our soils were characterized by highly diverse nutrient sources (litter, rhizodeposition), we expected high bacterial activity compared to typical bulk soils from lower depths (Marhan et al., 2011; Eilers et al., 2012; Müller et al., 2016). Rhizospheric soils contain large proportions of active bacteria (Blagodatskaya and Kuzyakov, 2013; Li et al., 2014), for which rRNA has been successfully used to analyze microbial communities before (Vieira et al., 2019). Moreover, previous studies of enzymatic potentials at our site demonstrated high and dynamic microbial activity (Regan et al., 2017). We hypothesized that ribosome maintaining communities in the topsoil of a modestly managed, homogeneously appearing grassland are environmentally regulated rather than stochastically structured, even in the absence of obvious habitat turnover. This hypothesis was extensively tested using spatio-temporal generalized additive models, variance partitioning, and a combination of phylogenetic and probabilistic null models.

The reader is invited to refer to the glossary and extended experimental procedures in the Supplementary Material for further explanation of design choices and terminology. For better readability, we use “community”, “abundance” and related terms below, but are always referring to communities and abundances of ribosomal reads.

## Materials and Methods

### Sample Acquisition

Soil samples were acquired from the SCALEMIC experiment, which was carried out within the frame of the ‘German Biodiversity Exploratories’ (Fischer et al., 2010), details of which are described in Regan et al., 2014. The site represents a temperate grassland soil, which is embedded in a larger homogeneous grassland region and which has been managed at low intensity, i.e., not fertilized, mown once per year, and grazed for only 1–2 weeks in late summer. At each of six sampling dates (April, May, June, August, October, and November 2011), 60 soil cores from the A-horizon (average sampling depth 10 cm) were taken from a 10 × 10 m physically homogeneous grassland plot, located 728 m above sea level in the Swabian Alb region (Germany). The site was subdivided into 30 equal subplots with 12 regularly arranged sampling locations in each, and with a minimum distance of 50 cm between each sampling location (Supplementary Section A). At each sampling date, two neighboring locations were sampled. No sampling location was used twice through the year. The site was unmanaged except for one mowing event (early August) and a brief period of sheep grazing in September. Two samples were lost; one in April and one in June, resulting in a total of 358 samples. Twenty-four environmental soil-related variables were measured for each sample, as described in Regan et al. (2014), for which PCA and box plot visualization is available in Supplementary Section A, and plant community coverage was determined in May, June, and October (Klaus et al., 2016). Previous analyses showed that this grassland plot features considerable spatio-temporal variability in plant productivity and diversity (Klaus et al., 2016), complex dependencies between general habitat properties and microbial parameters such as phospholipid fatty acid (PLFA) profiles and enzymatic activities (Regan et al., 2014, 2017), as well as between ammonia- and nitrite-oxidizing microorganisms (Stempfhuber et al., 2015). High turnover of arbuscular mycorrhizal fungi, likely driven by stochastic processes (Goldmann et al., 2019), and taxon-dependent influences of edaphic variables on protists (Fiore-Donno et al., 2019) were previously described as well.

### rRNA Extraction and cDNA Synthesis

RNA was extracted from frozen (−80°C) samples using a direct lysis protocol [modified after Lueders et al. (2004)]. For each sample, 600 mg of soil was transferred to a reaction tube, and cells were lysed by bead beating (45 s, 6.5 m/s). Nucleic acids were extracted using phenol/chloroform/isoamyl alcohol (c(v/v/v) = 25/24/1) and chloroform/isoamyl alcohol (c(v/v) = 24/1) and were precipitated from aqueous supernatants with two volumes of polyethylene glycol (30% w/v in 1.6 M NaCl) during centrifugation (90 min, 4°C). The nucleic acid pellet was washed with ethanol and resuspended in 30 μl elution buffer (Tris-HCl, 10 mM, pH 8.5). Nucleic acid concentration was estimated using a NanoDrop 1000 spectrophotometer (Peqlab Biotechnologie, Erlangen, Germany). DNA was removed from extracts by DNase treatment [1 U DNase I (1 U ^∗^ μl^–1^) per μg of nucleic acids, rounded up to the next multiple of 10; Fermentas/Thermo Fisher Scientific, Waltham, Massachusetts, United States)] RNA was precipitated with a 1/10 volume of sodium acetate (3 M, pH 5.2), washed with ethanol, and stored in RNAse-free water at −80°C. After RNA quantification using the Quant-iT RiboGreen assay (Life Technologies/Thermo Fisher Scientific, Waltham, United States), RiboLock RNase inhibitor (Fermentas/Thermo Fisher Scientific, Waltham, Massachusetts, United States) was added to each sample. For each sample, RNA was reversely transcribed into cDNA in two separate aliquots using GoScript (Promega, Mannheim, Germany) according to manufacturer’s instructions. RNA extracts were first mixed with random nucleotide hexamers (c (w/v) = 0.5 g ^∗^ μl^–1^), incubated for 5 min at 70°C, then quickly chilled on ice. Freshly prepared master-mix (consisting of reaction buffer, 25 mM MgCl_2_ 10 mM nucleotide mix, RNase free water, and reverse transcriptase (1 U ^∗^ μl^–1^) was then added to the sample. Samples were incubated for 5 minutes at 25°C, then 60 minutes at 42°C to enable reverse transcription, which was ended by increasing the temperature to 70°C for 15 min.

### Amplicon Library Construction, Sequencing, and Data Processing

We used the hypervariable region 3 of the bacterial 16S rRNA gene to create amplicon libraries for tagged sequencing with the Illumina HiSeq II system (Bartram et al., 2011). Briefly, primers contained the Illumina adapter sequence, the binding site for sequencing primers, and sequences for priming the target 16S rRNA region V3 [341f_wobble2; modified from (Muyzer et al., 1993)] and 515R (Lane, 1991), respectively. Additionally, the reverse primer included a barcode region of six nucleotides (represented by NNNNNN), yielding the following sequences: V3_F (5′-ATGATACGGCGACCACCGAGATCTACACTCTTTCCCTAC ACGACGCTCTTCCGATCTCCTACGGGWGGCWGCAG-3′) and V3_nR (5′-CAAGCAGAAGACGGCATACGAGATNNNNN NGTGACTGGAGTTCAGACGTGTGCTCTTCCGATCTCCGC GGCTGCTGGCAC-3′). The PCR was performed with the Phusion High-Fidelity DNA Polymerase kit (New England Biolabs, Ipswich, United Kingdom) following the manufacturer’s instructions, using 15 cycles of 94°C (15 s), 59°C (15 s), 72°C (15 s), after an initial denaturation step (94°C, 5 min), ended by a final elongation step (72°C, 7 min). Amplicons of the desired size were purified from gel with The NucleoSpin Gel and PCR Clean-up system (Macherey-Nagel, Düren, Germany). Sequencing was performed on the Illumina HiSeq platform with paired 100-base reads in three separate runs on multiple lanes each. Forward and reverse reads were first trimmed to 100 bp and dimers were filtered out based on detection methods implemented in FastQC.^1^ Reads were joined using fastq-join (Aronesty, 2013) at a minimum overlap of six nucleotides and allowing 20 percent mismatch. Joined reads were checked for chimeras by Uchime integrated in Usearch 5.2.3 (Edgar et al., 2011) applying the GOLD database from ChimeraSlayer^2^ as reference. After additional quality clean-up with CD-HIT [cd-hit-otu-filter.pl; Fu et al. (2012)] to remove ambiguous base calls or low quality sequences, the remaining reads were classified with the RDP-Classifier (Wang et al., 2007) for a first taxonomic overview covering the entire set of reads. From this dataset, operational taxonomic units (OTUs) were defined by mapping reads to a pre-clustered reference database [SILVA Ref NR 128 (Quast et al., 2013)] at 99% identity between query read and reference cluster with UCLUST within QIIME 1.9 (Caporaso et al., 2010). The closed-reference OTU clustering was the most appropriate choice, since the taxonomic resolution of the 16S rRNA is not constant across its various hypervariable regions. Global single- and doubletons, plastids, and mitochondria were removed from the OTU table, which in its final form covered 102,000,000 observations with high sampling completeness with respect to the assessed reference OTUs (Supplementary Section B).

### Data Analysis

Statistical analyses were carried out in R 3.5 and higher (R Development Core Team, 2018). Initial comparative β-diversity (i.e., between-sample diversity) analysis showed strong agreement between various data normalization and transformation approaches. The dataset was then scaled to total sample sums of all bacterial observations unless stated otherwise. If *P* values were generated, we used a significance level of 0.05, with adjustments for multiple tests according to the false discovery rate (FDR) method unless stated otherwise (Benjamini and Hochberg, 1995). In general, Mantel tests were used to quantify similarities between two distance matrices (999 permutations, Spearman rank correlation).

PERMANOVA, ANOSIM, and MRPP (Multiple Response Permutation Procedure) were calculated as initial omnibus tests to account for the influence of sampling date on community composition [package ‘vegan’, Oksanen et al. (2018)]. β-diversity was determined with abundance-weighted and unweighted UniFrac distances (Lozupone and Knight, 2005) and with Bray-Curtis dissimilarities in the framework of Legendre and De Caceres (2013), which partitions β-diversity into local contributions by site (“LCBD”). Significant LCBD was detected using 999 permutations with default *P* value adjustment [package ‘adespatial’, Dray et al. (2019)]. We also calculated the multiple site Sørensen dissimilarity and its nestedness and turnover components to account for abundance-unweighted (i.e., presence/absence-based) OTU turnover (package ‘betapart’, Baselga (2010)].

Variance partitioning with three separate distance-based redundancy analyses (dbRDA) was performed to decompose the explained community variance into its spatial, environmental, temporal, and joint components in package ‘vegan’. Spatial and temporal filters were proxied by distance based Moran Eigenmaps (dbMEMs) based on the sampling grid and sampling dates (Dray et al., 2006). We used forward selection of significant variables in the three dbRDAs (on Bray-Curtis dissimilarities) with a double stopping criterion (Blanchet et al., 2008) after a global significance test (Bauman et al., 2018). Function ‘varpart’ in ‘vegan’ was used with the Bray-Curtis dissimilarities of the OTU table, and the three forwarded selected variable sets.

α-diversity (i.e., within-sample diversity) was estimated with Hill-numbers *^0^D* and *^2^D* on the raw OTU table, which was intra- or extrapolated to the reference of twice the smallest sample size [package ‘iNEXT’; Chao et al. (2014)]. *^0^D* represents species richness with equal weight attributed to all species, whereas *^2^D* is the linearized form of the Simpson diversity index and emphasizes dominant species. *Post hoc* analyses to determine significant differences between sampling dates were performed by estimating marginal means (package ‘emmeans’, Lenth (2018)], with default *P* value adjustments and corrections for heteroscedasticity and spatial autocorrelation as described in Stempfhuber et al. (2015). The spatial variability within each sampling date was calculated as a coefficient of variation (CoV: standard deviation/mean), with global asymptotic and modified signed likelihood tests to identify significant changes in spatial variability throughout the year [package ‘cvequality’, Marwick and Krishnamoorthy (2019)].

Differential abundances of OTUs between specific groups of sites were identified using a consensus approach of different *post hoc* testing methods and test-specific data normalization procedures (Supplementary Section C). This was deemed necessary due to substantial differences between existing methods.

Whether local communities were shaped by stochastic and/or deterministic processes was assessed by first testing for significant phylogenetic turnover between communities (βMNTD; β mean nearest-taxon distance), from which the βNTI (β nearest-taxa index) was derived from a null model test of βMNTD (Stegen et al., 2013). This was followed by calculating pairwise Raup-Crick dissimilarities (RC_Bray_) between sites (Chase and Myers, 2011), weighted by OTU abundance (Stegen et al., 2013), after rarefying the OTU table to equal sampling depths. To quantify deterministic and stochastic influences on community assembly, we used the framework developed in Stegen et al. (2015) as illustrated in Feng et al. (2018). |βNTI| > 2 indicates that environmental selection is the primary assembly force, partitioned into heterogeneous (βNTI > + 2) and homogenizing selection (βNTI < −2). For |βNTI| < 2, a primary influence of stochastic effects was inferred. For this βNTI range, RC_Bray_ < −0.95 represents communities affected by homogenizing dispersal, whereas dispersal limitation (along with drift) results in RC_Bray_ > 0.95. For RC_Bray_ < |0.95|, moderate dispersal and weak selection results in an “undominated” scenario. The phylogenetic tree used for the βNTI/βMNTD estimation was obtained from SILVA^3^ which contained OTUs at 99% sequence similarity (“99_otus.tre”). The computing time necessary for the calculation of > 69,000 pairwise βNTI -comparisons lead to the decision to split the dataset by sampling date for the whole communities, and by the most abundant phyla and classes for calculations encompassing all 358 samples. For generation of abundance-based Raup-Crick diversity assessments, we used a re-implemented approach, which is much faster than previously used scripts (Supplementary Section D). While the original implementation in Stegen et al. (2013) calculated randomizations for each pairwise sample and then calculated Bray-Curtis distances,^4^ the new implementation achieves acceleration in two ways: (i) parallelization of the iterations and (ii) combined calculation of all pairwise samples using the faster *vegdist* function (package ‘vegan’) with underlying C + + code. The new implementation is available via GitHub^5^.

At the level of single OTUs, we fitted component-wise boosted generalized additive models (GAMs), which can accommodate the three effect classes present here in a single modeling framework: smooth effects (for environmental variables), linear effects (for sampling dates), and smooth spatial effects (including time-specific smooth effects for spatial effect estimates by sampling season). Only OTUs with at least 30 observations in the raw dataset were considered (arbitrarily set). For OTUs with > 40 zero observations, boosted GAMLSS (generalized additive models for location, scale and shape (Rigby and Stasinopoulos, 2005) were used under a zero-inflated negative-binomial distribution [package ‘gamboostLSS’, (Mayr et al., 2012; Hofner et al., 2015b)]. Otherwise, a negative binomial distribution was assumed, and boosted GAMs were fitted [package ‘mboost’, (Hofner et al., 2014; Hothorn et al., 2015)]. The choice of probability distribution was determined by initial out-of-bag estimates of prediction accuracy (not shown). To avoid overfitting, the optimal stopping criterion (number of boosting steps) was calculated via cross-validation by 25-fold bootstrapping. To obtain even sparser models, cross-validation was complemented with stability selection using 100 random subsamples (package ‘stabs’, Hofner and Hothorn, 2015; Hofner et al., 2015a). Variables were considered selected if they were present in more than 83% of the 100 models fitted on the 100 subsamples (controlled by the chosen Per Family Error Rate of 2). To compare the impact of spatial covariates on OTUs, we computed effect ranges (i.e., maximal – minimal effect) from the estimated OTU-specific spatial model. A higher effect range represents a larger impact on OTU counts. For more details on boosted GAMs, see Supplementary Section E.

## Results

### General Composition of the Potentially Active Bacterial Communities

Based on the RDP-classification and aside from unclassifiable bacteria (14.31% ± 1.27% SD, averaged across all samples), *Proteobacteria* (28.51 ± 2.8% SD) and *Actinobacteria* (24.48 ± 4.31% SD) together amounted to approximately 50% of an average community, followed by *Acidobacteria* (12.67% ± 1.28% SD). Other abundant phyla were *Planctomycetes* (8.34 ± 1.33% SD), *Verrucomicrobia* (6.02% ± 1.21% SD), *Bacteroidetes* (2.45% ± 0.84% SD), and *Firmicutes* (1.76% ± 0.54% SD). All other phyla contributed less than 1% of all reads each. *Acidobacteria* Subgroup 6 and *Planctomycetaceae* emerged as the most common families across all samples. Twenty-two percent of all reads could be mapped to reference taxonomy, yielding 16,944 reference OTUs, of which 1398 resident OTUs were ubiquitously detected in all samples. This group alone represented 89.8% of all reads in this OTU dataset. Another 9133 transient OTUs occupied less than 10% of all sites. A highly significant log-log relationship between abundance and frequency was found at the OTU level (*P* < 0.0005), which was conserved between phyla (Supplementary Figure S1) and revealed a steady abundance to frequency ratio of between 10 and 1 reads per sample. In agreement with the RDP-classified dataset (encompassing all reads), alphaproteobacterial and actinobacterial lineages dominated within the 22 most abundant OTUs [minimum mean relative abundance to total bacterial observations > 0.1% (Supplementary Table S1)].

### Spatio-Temporal Variability of Bacterial α- and β-Diversity

A significant influence of sampling date on community composition was attested by three omnibus tests (OTU level; PERMANOVA, ANOSIM, MRPP, *P* < 0.001 in all cases). Visualization of this influence with ordination techniques (UniFrac/PCoA and Bray Curtis/NMDS) revealed a clustering that was strongest for April and for a group of communities sampled in June (Supplementary Figure S2). We calculated the local contribution of each community to β-diversity (LCBD), and found 66 of the 358 stations (18.4%) exhibiting significantly differing community compositions, most of which occurred in the April (n = 26), and June (n = 15) samplings (Supplementary Figure S3). After the June sampling, the average LCBD per sampling date did not fluctuate significantly (Table 1 and Supplementary Figure S4). On the three dates for which plant coverage data was available (May, June, October), no relationship between plant and bacterial β-diversity was found (Mantel tests, UniFrac and Bray Curtis distances for bacteria vs. Bray Curtis and Hellinger distances for plant coverage, *P* > 0.05 in all cases). We used the variance partitioning approach to disentangle the single and joint effects of space, time, and environment. 35% of the observed variance in β-diversity could be explained by three separate, forward selected dbRDAs. We found pure effects of space (9%), soil variables (9%) and time (2%). Soil variables were interacting with space (Soil∩Space = 7%) and with time (Soil∩Time = 5%), while space and time (Time∩Space) did not interact. Another small fraction of the variance was explained by all variable sets acting together (Soil∩Space∩Time = 2%). Soil moisture and pH were the most significant environmental variables (Supplementary Table S2), followed by EOC, C_mic_, N_mic_, Clay content, phosphate and litter mass.

**TABLE 1:** Temporal and spatial variability of α- and β-diversity measures in the SCALEMIC plot.

Sampling Season	Mean LCBD	SD	Significance Group (# divergent sites)	CoV	Global tests for CoV Significance:
April	0.0037	0.0016	a (26)	0.444	*P* < 0.001
May	0.0023	0.0016	b (7)	0.716	
June	0.0040	0.0046	a (15)	1.156	
August	0.0025	0.0010	b (8)	0.387	
October	0.0021	0.0008	b (4)	0.367	
November	0.0022	0.0010	b (6)	0.456	

**Sampling Season**	**Mean *^0^D***	**SD**	**Significance Group**	**CoV**	**Global tests for CoV Significance:**

April	4774.2	174.3	a	0.036	*P* < 0.001
May	4739.6	264.3	ab	0.055	
June	4659.4	246.5	bc	0.052	
August	4454.5	202.7	d	0.045	
October	4613	166.3	c	0.036	
November	4624	177.6	c	0.038	

**Sampling Season**	**Mean *^2^D***	**SD**	**Significance Group**	**CoV**	**Global tests for CoV Significance:**

April	351.4	58.9	a	0.168	*P* > 0.05, not significant
May	281.4	45.1	b	0.160	
June	268.2	47.0	b	0.175	
August	278.7	40.9	b	0.147	
October	250.2	33.3	c	0.133	
November	244.6	38.1	c	0.156	

*Shown are means and standard deviations of the local contribution to β-diversity (LCBD), of species richness (Hill number 0, *^0^D*), and of the linearized Simpson diversity (Hill number 2, *^2^D*). Spatial variability is represented by the coefficient of variation (SD/mean). Global significance tests of spatial variability changes between sampling seasons include asymptotic and modified signed likelihood tests. “# divergent sites” in the top table refers to the number of sites with statistically significant contribution to overall β-diversity. A boxplot depiction of these values is found in Supplementary Figure S4.*

We then tested whether the emerged patterns of β-diversity were caused by species turnover (i.e., replacement) or species loss (i.e., nestedness). In contrast to other β-diversity measures, this presence/absence based measurement of β-diversity was high, without significant changes between samplings (0.84 < β_S__ø__r_ < 0.87). Only turnover, but not nestedness, contributed to respective multiple site dissimilarities. Partitioning the OTU table by decreasing frequency showed that the detected high species turnover was almost entirely attributable to the rare biosphere, and that total β-diversity was low, while nestedness and turnover were balanced when only resident and abundant OTUs were considered (Supplementary Figure S5).

Moving from the community level to specific populations, distinct temporal abundance profiles were detectable at all taxonomic levels (Supplementary Figures S6–S8, for phylum and subgroup level seasonality based on the RDP-classified data, and for abundant OTUs). A strong replacement of *Proteo*- by *Actinobacteria* between April and May dominated all other observed population-level changes. This major turnover was driven by a substantial decrease in beta- and delta-, but not alphaproteobacterial abundance. Accordingly, abundant, resident OTUs experienced a significant abundance change between these two months. Other major groups featured mostly homogeneous distributions across the year (e.g., *Spartobacteria*, *Gammaproteobacteria*, *Acidobacteria* Subgroups 3 and 17). In contrast, spatial variability exceeded the differences between monthly abundance means for many other major groups, e.g., phyla *Nitrospirae* and *Planctomycetes*, and classes *Spartobacteria*, *Acidobacteria* Subgroup 17, and *Gammaproteobacteria*.

The two α-diversity measures showed distinct temporal patterns (Table 1 and Supplementary Figure S4). Compared to the diversity of abundant species (expressed by the linearized Simpson diversity), which strongly peaked in April and significantly declined until winter, species richness slightly decreased toward summer, when it reached an annual low in August after which it increased again. The within-month spatial variability (CoV) of the Simpson diversity was generally higher than for species richness but did not change significantly between sampling dates, in contrast to species richness, which had lower but significantly oscillating values across time (Table 1). Species richness was positively influenced by carbon pools (SOC and EOC; selected in all 100 stability selected GAM subsamples, but only after the time variable was removed from the model), at moderate correlation strength (in a linear framework, the obtained Pearson correlation coefficients ρ were 0.33 for EOC, and 0.22 for SOC, respectively; *P* < 0.01 in both cases). By contrast, Simpson diversity was strongly correlated to soil moisture (100% of GAM subsamples, corresponding to Pearson’s ρ + 0.47, *P* < 0.001 in a linear framework).

### Spatio-Temporally Isolated Community Shifts

**FIGURE 1:**
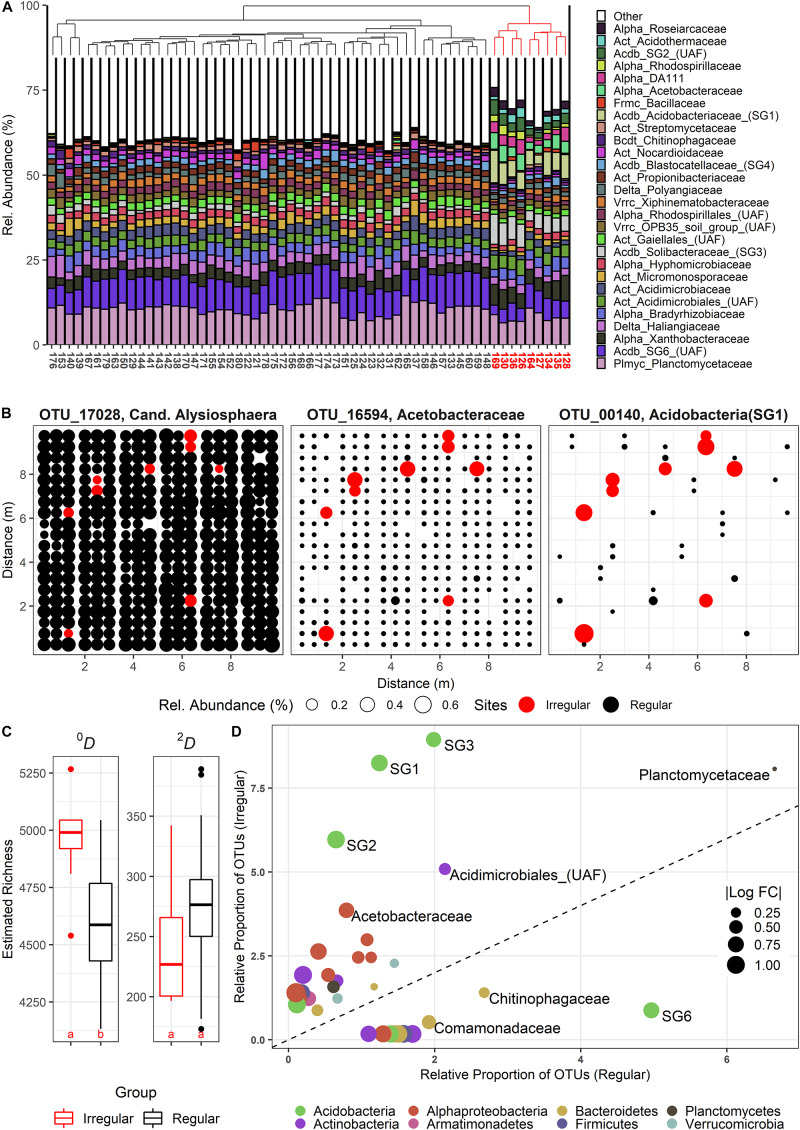
(A) Composition of bacterial communities at the family level during the June sampling campaign, clustered by complete linkage. Irregular, strongly deviating communities are indicated in red. **(B)** Spatial bubble plots of three selected OTUs (from left: resident, but significantly less abundant in irregular communities (red); resident, but highly stimulated in irregular communities; rare, and highly stimulated in irregular communities). Each circle represents one site in the SCALEMIC experiment across the entire year. Circle size correlates with relative abundance. **(C)** Boxplots showing α-diversity in irregular (red) vs. regular sites (black). Species Richness (Hill number 0, *^0^D*) and the linearized Simpson diversity (Hill number 2, *^2^D*) are depicted. Letters indicate groups of statistically significant differences (estimated marginal means). **(D)** Relative shifts of abundance-unweighted OTU proportions between the two community types, with no change represented by the dashed line. Families above this line feature significantly increased OTU frequencies in the irregular communities. Bubble sizes indicate the absolute of the log10 fold change between the relative OTU frequencies in irregular vs. regular communities (and vice versa, below the dashed line). UAF = Unassigned family within the given clade.

The sampling in June yielded several sites with community compositions that significantly deviated from all other stations. A Bray-Curtis based complete linkage clustering of the June communities assigned nine sites to a group of irregular, but similar communities (Figure 1A) in two loosely parallel bands across the plot (Figure 1B), indicating a uniform shift toward an alternative community state. We classified 570 OTUs as differentially active (“DA”) in the irregular sites, among which we found both (almost) unique OTUs and intermediate forms (Figure 1B). DA OTUs were concomitantly present with the common set of resident species, resulting in significantly elevated *^0^D*-richness levels (Figure 1C). By contrast, *^2^D*-richness differences were not altered, although DA OTUs were frequently among the most abundant OTUs in the irregular sites. Thus, the elsewhere dominant, resident OTUs were sometimes outcompeted [e.g., OTU 17028 (Figure 1B)]. Within the set of 570 DA OTUs, a strong taxonomical shift toward distinct alphaproteo-, actino-, and acidobacterial families, but also toward *Armatimonadetes* was found (Figure 1D). We initially could not detect any environmental explanations for the community shifts with multivariate and/or linear analyses. However, GAMs fitting additional plant-derived data for the June data set (Klaus et al., 2016) frequently reported a positive effect of *Dactylis glomerata* subplot coverage at intermediate total plant coverages and lower plant evenness (Supplementary Figure S9).

### Quantifying Stochasticity and Determinism in Local Community Assembly

**FIGURE 2:**
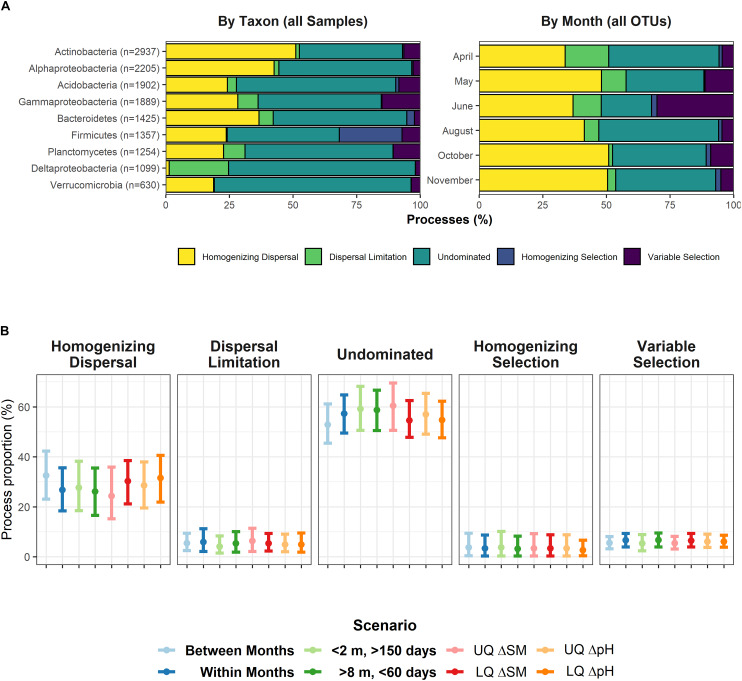
**(A)** Assembly processes for nine major bacterial groups (left panel) across all 358 samples and for the six individual sampling dates (right panel), encompassing between 59 or 60 samples. *Gammaproteobacteria* include *Betaproteobacteria*. **(B)** Assembly processes in defined scenarios: Temporal distance > 0 (“Between Months”), Temporal distance = 0 (“Within Months”), spatial distance < 2 m & temporal distance > 150 days, and spatial distance > 8 m & temporal distance < 60 days. The latter two scenarios theoretically support homogenizing dispersal, and dispersal limitation, respectively. UQ/LQ = Upper/lower quartiles of between-sample differences in soil moisture (SM) and pH. Depicted is the mean with its 95% confidence interval for values averaged over nine major bacterial groups (see left panel of **A**). No pairwise comparison yielded a significant difference of distribution means.

Due to heavy computational burden, we decided to split the dataset in two different ways: i) by major taxonomic groups (phylum or class, > 600 OTUs) for the entire set of 358 samples and ii) by sampling date (all OTUs, 59 or 60 samples each). In almost any case, undominated scenarios, e.g., the interaction between deterministic and stochastic processes explained most of the assembly. However, we also found evidence for strong stochastic assembly processes acting alone (Figure 2A). At each time point, homogenizing dispersal explained between 34% and 51% of the assembly mechanisms, while undominated scenarios characterized between 19% and 47%. For individual populations, dispersal was never limited, but acted as a homogenizing agent, as well, except for *Deltaproteobacteria*. By contrast, if selection played a role, it rather worked as a heterogenizing agent, as variable selection dominated over homogenizing selection (except for *Firmicutes*).

We also tested whether or not the process patterns were influenced by spatial, temporal or important environmental gradients. We split the dataset into several scenarios (“Within Months”, “Between Months”, “High spatial and low temporal distance” (conceptually facilitating dispersal limitation), and “Low spatial and high temporal distance” (conceptually supporting homogenizing dispersal), and low and high differences in soil moisture and pH, respectively) to determine whether process patterns changed within these scenarios. However, we found no evidence for any scenario to influence assembly processes (Figure 2B).

As previous analyses had pointed to a highly dynamic rare biosphere, we finally looked for similar effects within the βNTI/RC_Bray_ framework as well. To determine this, we randomly drew 10 subsets of 500 OTUs (with 120 – 240 non-zero observations) belonging to the rare to intermediate biosphere and found that these subsets were largely controlled by dispersal limitation (61.4 ± 1.1% SD) and undominated process interactions (31.1% ± 0.4% SD).

### Spatio-Temporal and Environmental Controls of OTU Abundance

By using boosted GAMs and GAMLSS, we decomposed effects on OTU abundance into interpretable partial effects by environmental, spatial, and temporal variables (Supplementary Table S3). 9553 OTUs with more than 30 observations in the entire data set were individually modeled and grouped at the genus level if possible. Cross-validated models selected significantly more candidate variables than the very conservative stability selection. Both variable selection methods agreed in assigning space (selected in 82% of all 9553 cross validated models, 69% when stability selected), pH (60%/25%, respectively), time (57%/21%), and soil moisture (57%/19%) as the most frequently selected variables. Relative selection frequencies were low using the stability selection approach, but even the least often selected predictors (e.g., protozoal PLFA) were picked with high confidence in no fewer than 110 out of all models. *Acidobacteria*, *Deltaproteobacteria*, *Planctomycetes*, and *Chloroflexi* clades responded to pH most frequently, whereas many *Bacteroidetes*, *Actinobacteria*, and especially *Firmicutes* affiliated groups did not. Soil moisture was best fitted to several actinobacterial groups (e.g., genera *Iamia*, *Nocardioides*, or *Solirubrobacter*). High individual selection frequencies among groups with high OTU numbers included nitrate (genera *Massilia*, 26.4%), C/N ratio (genus *Chthoniobacter*, 25.4%), clay content (order *Tepidisphaerales*, 28.1%), and bulk density (phylum *Latescibacteria*, 34.1%).

**FIGURE 3:**
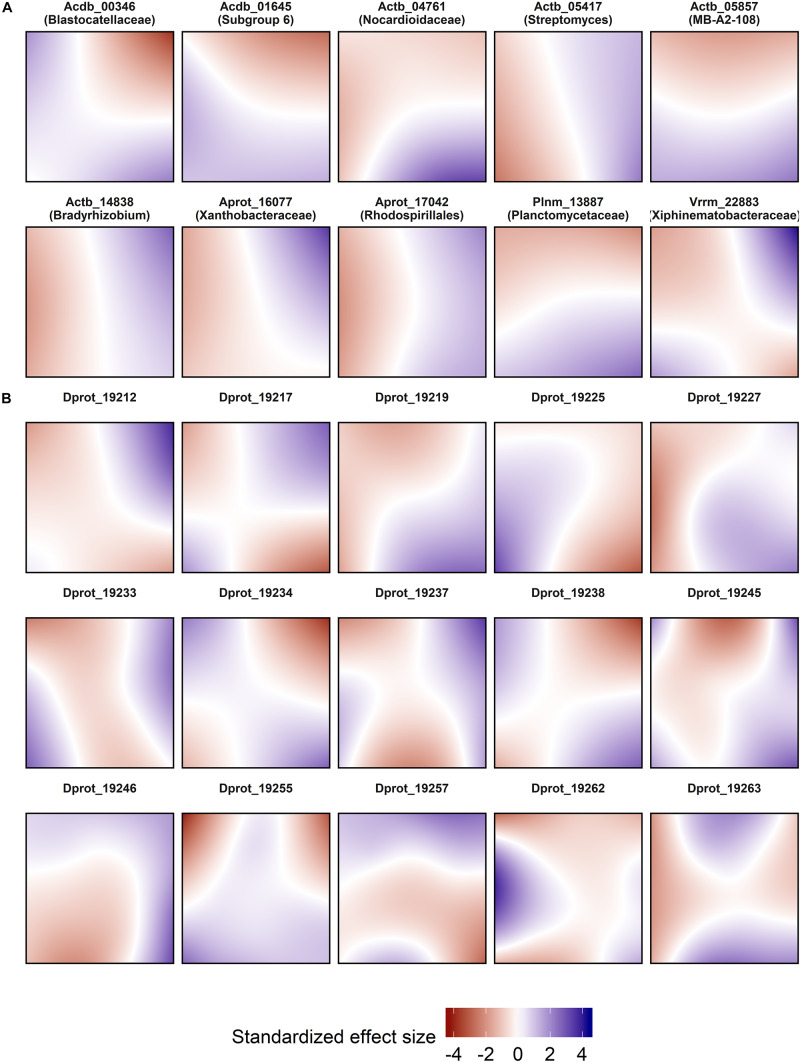
Spatial abundance models of selected OTUs. **(A)** Highly abundant OTUs. **(B)** Phylogenetically close OTUs within genus *Bdellovibrio* with decreasing abundance from top left to bottom right. Each panel represents a map of the SCALEMIC experimental site with the top pointing to the north direction, and shows the pure spatial model *prediction* for a single OTU. Blue areas represent areas in which the spatial effect predicts high local abundances. The maps encompass the effects of all sampling dates after adjusting for temporal and environmental variables in the same model. For each model, abundance data from 358 sampling locations were smoothed using bivariate P-splines with a grid of 24 × 24 knots. The partial effects of the models were individually centered and scaled (mean = 0, SD = 1).

**FIGURE 4:**
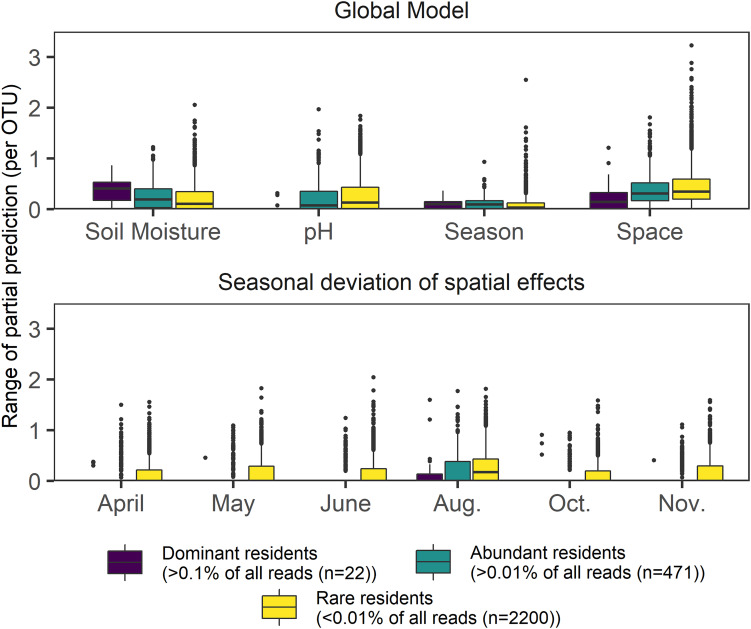
Boxplots showing the response range of partial effects assigned by GAMs to the four most frequently picked variables in a global model encompassing space, season, and environmental variables. The dataset was separated by relative abundance and includes 2693 OTUs, which were modeled using negative binomial GAMs; lower panel depicts temporal deviations from the main spatial effect found in a global model. Effect size values were not scaled.

Spatial models were used for abundance prediction (Figure 3), and included pure effects of location decomposed from time and environment. The effect size ranges of the spatial predictor were comparable to the most effective edaphic parameters (pH and soil moisture; Figure 4). Model complexity increased with decreasing OTU abundance, and could vary significantly within phylogenetically close relatives [exemplarily shown for bacteriolytic *Bdellovibrio* (Figure 3B)].

We then checked whether or not pure spatial effects changed if sampling dates were assessed individually, on a subset of 2693 resident OTUs (present in at least 320 samples), (Figure 4). The 22 most dominant OTUs (mean relative OTU abundance > 0.1% each) showed the lowest seasonal deviations from the main annual effect. Though decreasing OTU abundance increased the likelihood of spatio-temporal variability (indicated by increasing effect deviations, Figure 4), the main annual effect always had the highest average range in general for all three subsets of the resident OTUs (dominant, abundant, rare). High seasonal variability of the spatial effect size was found for rare resident OTUs during all months, but highest spatial variability overall was evident in August. Soil moisture and pH effects yielded opposite patterns on rare, abundant, and dominant resident OTUs. Within the dominant OTUs, soil moisture was observed to have the strongest effect, whereas pH and space better explained profiles of less abundant residents. Pure seasonal effects did not show rarity-specific effects.

## Discussion

### General Dynamics and Assembly of Local Communities

We monitored bacterial rRNA abundances at 358 stations on a 10 m x 10 m plot, equally distributed across six inter-annual sampling dates, and expected that those bacteria maintaining transcriptional potential would be subjected to environmental filtering rather than to stochasticity. In contrast, we found that both process types together shaped the local communities, which in total resulted in a strong community homogenization. Time and space were both important predictors of diversity and abundance of bacterial rRNA counts. Moreover, our analyses pointed to a stable but oscillating community backbone of resident OTUs beyond which a rare biosphere showed higher spatio-temporal variability and turnover.

Three approaches were used to account for assembly principles: i) βNTI/Raup Crick modeling revealed a high proportion of stochasticity acting alone, which however also often interacted with deterministic process. This was supported by ii) variance partitioning, which found both space and environment equally impacting the community. Finally, we could use iii) spatial GAMs to map the OTU-level consequences of the identified assembly processes and find a rarity-dependent effect of space. Moreover, both variance partitioning and GAMs agreed in finding the most important individual variables, filling a conceptual gap of the βNTI/Raup Crick approach, which was not designed to identify the environmental filters at work.

Almost 1400 OTUs (representing ∼90% of all reads) in our site were sufficiently adapted to the present environmental gradients to maintain ribosomes at any time and in any location. This conclusion is supported by the finding that substrate pools (soil organic carbon, total nitrogen, EON/EOC) were of lesser importance for OTU abundance and changes in β-diversity in our most parsimonious models, compared to soil moisture and pH. We also did not find strong evidence for correlations between bacterial and plant β-diversity or of a strong influence of plant-derived variables on OTU abundance. Feng et al. (2018) explained that the transition to purely deterministic species sorting happens when environmental changes occur outside tolerated gradients. It seems likely that in our grassland those thresholds were never exceeded, at least not for the resident OTUs. Accordingly, spatial GAMs, and total site dissimilarity analysis agreed in assigning the largest effect sizes to the rare biosphere, which has been demonstrated in other habitats as well (Gobet et al., 2012; Kim et al., 2013).

The finding that the assembly patterns for most tested populations or time points were consistently dictated by either homogenizing dispersal or a combination of (weak) deterministic and neutral processes aligns well with the conceptual framework of Langenheder and Lindström (2019) for small observational scales, and contrasts with the detection of habitat-related bacterial turnover at larger scales (Powell et al., 2015). We can also confirm recent results describing that stochasticity rather than taxonomic composition facilitates variability of soil bacteria (Albright et al., 2019a), and our results are consistent with reports that grasslands promote dispersal of bacteria below- and above-ground (Albright and Martiny, 2017; Goss-Souza et al., 2017), possibly due to lack of pronounced spatial nutrient variability (Curd et al., 2018). Two major bacterial groups differed in their dispersal ability: *Deltaproteobacteria*, consisting mostly of *Myxococcales*, faced significant limitations in their ability to stochastically disperse, probably due to their multicellular, biofilm-based lifestyle (Muñoz-Dorado et al., 2016.). Firmicutes, however, were found to be homogenized by environmental selection rather than dispersal, owing to the fact they were often phylogenetically less diverse than predicted by chance (βNTI < −2). Finally, the June communities experienced the highest influence of variable selection, thanks to the presence of the irregular communities. Besides, the relative contribution of each process type remained remarkably constant across phylum/class level populations and across individual time points, with no evident impact of space, time or important environmental gradients. Taken together, the detected assembly principles at our site allow the interpretation that an apparent absence of physical barriers in the soil resulted in homogenization of an intact community that had been established long before the experiment. Interestingly, a similar experiment with dryland soils featuring a mosaic of crusts and plant cover contrastingly showed high β-diversity on very small scales, driven by habitat turnover (Albright et al., 2019b).

### Interacting Processes Affect Spatio-Temporal Variability

In general, soils are regarded as the habitat harboring the most temporally invariant microbial communities (Shade et al., 2013), likely resulting from the many micro-habitats present in soils at very small scales (Tecon and Or, 2017), which in theory should limit the rate and success of independent dispersal processes (e.g., Yan et al., 2019). However, reports of soil microbial communities with pronounced temporal variability related to season, soil, and management type are accumulating (Lauber et al., 2013; López-Mondéjar et al., 2015; Zifcakova et al., 2016; Albright et al., 2019b; Landesman et al., 2019). In our case, the sampling season significantly structured α- and – to a lesser degree – β-diversity and resulted in specific abundance patterns across all taxonomic levels. Unlike other natural grasslands (Barboza et al., 2018), abundance-weighted β-diversity did not show a strong clustering by time, as the clearest separation in ordination was found between April and all other time points (Supplementary Figure S2), which matched significant environmental changes between April and May (Supplementary Section A.3) and changes in nutritional limitation of plants (Klaus et al., 2016). This environmental turnover drove huge phylum-level abundance shifts (along with a significant drop in Simpson diversity, overall β-diversity, and cell numbers), similar to simulated wetting/desiccation experiments (Barnard et al., 2013). After rewetting of the soils due to heavy rainfall (in combination with the removal of plant biomass by mowing and significant drops in EOC/EON pools) before the August sampling, species richness was at a seasonal low at the same time that many dominant OTUs exhibited a relative abundance peak. Although soils had significantly dried out again by October, the associated community changes were not nearly as drastic as those between April and May, indicating different, possibly stabilizing mechanisms at work later in the year. In fact, apart from the irregular sites in June, no significant change in average LCBD per month was detected at all after spring.

Our most conservative additive models predicted that deterministic processes were mostly driven by pH – despite a moderate range of 1.25 pH units (5.98 – 7.23) – and soil moisture, which was confirmed by community level modeling (dbRDA). Both variables have previously been identified as major factors that directly or indirectly shape soil microbial communities (Brockett et al., 2012; Cruz-Martínez et al., 2012; Maestre et al., 2015; Lammel et al., 2018; Tripathi et al., 2018; Lupatini et al., 2019). pH seems to play a mediating role in our grassland, as on the one hand it has been shown in other systems to facilitate stochastic processes at near neutral range (Tripathi et al., 2018), while on the other hand it is the likely candidate variable being responsible for the weak deterministic processes at our site when assembly was undominated. Soil moisture may rather act as an enabler of stochasticity. Water availability (and soil structure) is a natural limitation to undirected dispersal of microbes (Tecon and Or, 2017); however, by connecting microhabitats, it also promotes biotic interaction, and therefore may confound the detection of stochastic effects. Intriguingly, we found the strongest dispersal limitation in April, when soil moisture (among other variables, including total cell numbers) peaked. This well demonstrates the ambiguous impact of water-connected habitats, which not only enables movement but also increases interaction, and thus competition among dispersers. Likewise, many abundant OTUs were least dominant when soil moisture was high, inducing a seasonal peak of Simpson diversity, i.e., a diversification of dominant species. The probabilistic framework used here addresses this possible caveat by assuming that stochastic assembly is only possible if phylogenetic turnover is not drastically different from chance alone, and thus not driven by environmental gradients (including structuring variables, e.g., moisture). Although soil moisture and bulk density values showed seasonal fluctuations [and were in some cases correlated with space itself (Supplementary Table S4)], our results do not imply that these soil structure variables were strongly disturbing or promoting stochastic assembly. To this end, rare OTUs were most likely influenced by dispersal limitations, but soil moisture exerted less control on them (Figure 4).

Spatial distributions of microbes at the plot scale have been assessed in some other studies (Keil et al., 2011; Wang et al., 2012; Mukherjee et al., 2014) but their temporal stability has been less frequently examined (Regan et al., 2014, 2017). Here we show that spatial variability depends on abundance, as well demonstrated for *Bdellovibrio*. Moreover, a large proportion of the most abundant OTUs had a remarkably consistent spatial distribution, in complementary patterns (*Actinobacteria* OTU 05857 vs. *Planctomycetes* OTU 13887) as well as in congruence (*Acidobacteria* OTUs 00346 and 01645). In all but one case (*Verrucomicrobia* OTU 22883), the dominant OTUs exhibited clear unidirectional spatial effect gradients. Our spatial prediction models also show that pure spatial effects do not act homogeneously across the plot, corroborating the varying degree of undominated vs. stochastic assembly ratios. Moreover, the relation between decreasing abundance and increasing patchiness of the spatial models corroborates the finding that rare populations were largely controlled by dispersal limitation and experienced the largest effect sizes of space (Figure 4).

Finally, we also extracted additive models partitioned by environmental variables and gained effect estimate profiles from thousands of individual OTUs (Supplementary Figure S10, for pH, soil moisture and litter). Although discussing them in detail is beyond the scope of this study, our data provide the opportunity to examine whether OTUs exhibit phylogenetically regulated functional niche effects or instead form a redundant web of ecologically similar organisms (Konopka et al., 2015; Mandakovic et al., 2018). The similar shape and position of many partial functions, especially in ranges of environmental variables that support mesophiles, suggest overlapping niches of co-existing OTUs. Interestingly, the obtained curve arrays were remarkably conserved across phyla, except for partial effect models for soil moisture of *Acidobacteria*. Accordingly, strong taxonomic signals were also absent when assessing assembly principles at the population level.

### Isolated Local Community Shifts

The heterogeneous matrix of soil is known to harbor heterogeneous communities at very small scales (O’Brien et al., 2016), but our study shows that fine-scale heterogeneities were undetectable with the size of cores and scales we used for sampling. Still, the unexpected finding of very unusual community composition during the June sampling is a main highlight of our study.

Although our data did not provide a definite explanation of the causes of the community shifts, several scenarios are possible. The rise of the irregular communities could have been triggered by priming effects caused by uncommon substrates (e.g., by defecation of transient grazers) resulting in shifts in nitrogen availability; this could have consequently favored either stoichiometric growth by opportunists (if N-availability was high) or nutrient mining K-strategists (if N-availability was low) (Fierer et al., 2012; Di Lonardo et al., 2017). The C/N ratio was on average higher in the irregular sites, although this difference was not statistically significant. It also seems unlikely that we coincidentally observed a short-lived invasion triggered by, e.g., undirected transport via animals. Differentially active OTUs apparently were natural members of the metacommunity, as they were evident in other sites and at other sampling dates as well, either in traces or even ubiquitously (but not nearly as abundant, which could indicate resting cell states). And finally, given that cell counts in these communities were high but not significantly different from bulk communities, it is difficult to characterize these events as soil blooms, which have been described in association with fertilization or contamination (Udikovic-Kolic et al., 2014; Fuentes et al., 2016), but never in unmanaged grasslands.

Instead, our models suggest that the community switches were driven by above-ground vegetation parameters and possibly influenced by the presence of the common grass *Dactylis glomerata*. Stages of plant growth also influenced fluctuations in soil nutrient concentrations, perhaps creating competition with microbes for resources (Regan et al., 2014, 2017). Thus, their classification as hot moments (Kuzyakov and Blagodatskaya, 2015) is tempting, as these events are often facilitated by labile organic inputs from plants; and as our A-horizon soil was strongly affected by the dense root system of its grassland cover. Although the stimulated taxa have not been described as rhizosphere-specific, copiotrophic, or as competitors (Ho et al., 2017), e.g., capable of quickly responding to nutrient pulses, recent research has revealed that, e.g., subgroup 1 *Acidobacteria* (which were dominant in the irregular communities) support feedbacks between distinct communities in the rhizosphere and its surrounding soil (Kielak et al., 2016; Kalam et al., 2017).

It is clear that irregular communities soon returned to a state representative for this grassland in previous (and succeeding) sampling events. If we assume that external or internal disturbances drove the switches, the observed full recovery demonstrates a strong capacity of the studied communities to return to a stable state (Shade et al., 2012; Faust et al., 2015). It is possible that the overall lack of dispersal limits enabled the resident OTUs to reestablish once the conditions leading to the irregularities were gone. In our case, a strong external disturbance (the almost complete removal of above ground plant biomass by the August mowing) might even have terminated – not caused – those conditions, which favored the alternative community states. Nevertheless, it was not possible to infer how long the irregular communities would have prevailed or if more sites would have been affected under undisturbed conditions.

Future implications of these surprising findings for sampling designs depend on *a priori* knowledge about sampling areas and on available labor, but as sequencing costs are constantly decreasing, the analysis of single cores (instead of the often applied pooling of 3 – 5 cores per plot) may yield surprising heterogeneity.

### Limitations and Caveats

Phylogenetic uncertainties of the hypervariable region 3 of the 16S rRNA required us to omit those reads which were not 99% similar to already known sequences, which led to a loss of the remaining signals. However, our dataset is still extraordinarily large, and given the overall homogeneity of the results, we believe that extrapolation to the entire community, e.g., as identified with a 97% threshold for *de novo OTU* clustering, is justified. Preliminary analysis with a dataset comprising of non-clustered, RDP-classified reads found the same trends of β-diversity as presented in this manuscript, including the irregular communities during the June sampling (Richter-Heitmann, 2016).

Two other limitations of our analysis should be considered. First, “space” and “time” may be masking environmental gradients we did not measure, and thus do not completely represent stochastic processes. Most, if not all, studies of spatio-temporal distribution patterns of microbes must deal with this issue. Hence, the now well-established framework of combining βNTI and Raup-Crick null models is critical to our approach, as it determines whether there is any indication of stochastic assembly in the first place. In our case, we have good evidence to assume that “space” and “time” are indeed proxies for stochasticity, as environmental selection – as an independently acting process – did not play an important role. We found significant effects of space and time on OTU abundances, local community composition, which increased for the rare biosphere. Moreover, in both modeling approaches (boosted GAMs and dbRDA) – although conceptually being very different – we found strong evidence for the influence of the location on OTU abundance and community composition. However, clear quantification is still very difficult, and we feel that the effect decomposition provided by GAM boosting is helpful in finding “pure” effects, while dbRDA provides insight to the joint variability which is best explained by spatially or temporally dependent environmental gradients.

Another caveat of our study is that neither the analysis of rRNA genes nor rRNA transcripts can exclude cells in (short-termed) resting states. Regardless of the marker, dormant cells could be problematic for assembly assessments insofar as they do not proliferate (which can be deterministically or stochastically regulated) or actively move (e.g., along nutrient gradients). However, unlike DNA signals, which are often contaminated with old relic residues (Carini et al., 2016), rRNA reflects past, present, and future protein synthesis potential (Blazewicz et al., 2013), and is therefore better suited than rDNA to reflect the potential of populations to establish themselves within a community. Since our analysis likely included cells in any activity state, residency could theoretically be explained at least in part by dormancy. However, it should be considered that i) our soils represented rhizosphere or rhizosphere-affected soils, which are characterized as activity hot spots (Blagodatskaya and Kuzyakov, 2013), ii) previous experiments showed high microbial activity in the same soil (Regan et al., 2017), iii) bacterial cell counts and 16S rRNA gene copy numbers showed considerable seasonal variability (Regan et al., 2014, 2017; Stempfhuber et al., 2015). The studied site was specifically selected, as unfertilized perennial grasslands with high plant diversity have been shown to have higher soil organic carbon, total nitrogen, and microbial carbon; greater food web complexity, more complex biological communities (Grayston et al., 2001; Culman et al., 2010), and to use nitrogen more efficiently than those with less plant diversity or more intensive management such as croplands, especially in nutrient-limited soils (Zak et al., 2003; Kleinebecker et al., 2014). Moreover, our results show that homogenizing dispersal was much more important than variable selection in the assembly of the local communities, and passive dispersal of dormant cells can easily be integrated in such a scenario.

## Conclusion

We combined a unique spatio-temporal sampling design with high-resolution molecular tools and sophisticated analysis to identify the main drivers of the assembly of potentially active bacteria at the plot scale. Our central questions asked whether intra-annual changes in microbial activity potential can be detected on small spatial scales with intensive sampling efforts and whether they would follow stochastic or deterministic principles. The emerging picture from this study was that of many concomitantly present OTUs, whose individual small scale biogeographies combined to create a dense system throughout the A-horizon of this grassland.

Considering all our results, we conclude that spatio-temporal variation can be partitioned into an oscillating core microbiome and a dynamic rare biosphere which was more likely subjected to species turnover and spatio-temporally explicit absence. We found that neither stochastic nor deterministic processes dominated over community assembly, but that in many cases unlimited dispersal overpowered selection and acted as a homogenizer of local communities, whereas the rare biosphere rather experienced dispersal limitation.

Finally, even though the communities showed strong spatio-temporal stability, there was the potential for short-lived and spatially isolated community shifts. The observation of bloom-like events at the plot-scale emphasizes the importance of frequent sampling over space and time to ensure that observed communities are representative of composition states.

## Data Availability Statement

The datasets generated for this study can be found in the Short Read Archiv, Project-ID PRJEB10957, http://www.ebi.ac.uk/ena/data/view/PRJEB10957.

## Author Contributions

EK and SM designed the SCALEMIC experiment. TR-H extracted nucleic acids, generated sequencing libraries, and carried out all statistical analyses, together with BH. BH designed and provided the general additive modeling framework. F-SK provided scripts for the determination of community assembly principles. BB, SH, PW, and JS provided parts of the bioinformatics pipeline for downstream analysis of sequence data. JS, PW, JO, and MF developed the concept of the current study. TR-H, BH, and MF drafted the manuscript. RB, KR, DP, DB, and SM provided data. KR edited and corrected the manuscript’s English. All authors read and revised the manuscript.

## Conflict of Interest

The authors declare that the research was conducted in the absence of any commercial or financial relationships that could be construed as a potential conflict of interest.

## Footnotes

1 http://www.bioinformatics.babraham.ac.uk/projects/fastqc/

2 http://drive5.com/otupipe/gold.tz

3 https://www.arb-silva.de/download/archive/qiime/

4 https://github.com/stegen/Stegen_etal_ISME_2013

5 https://github.com/FranzKrah/raup_crick

## Supplementary Material

FIGURE S1 Log10 transformed frequencies (sampling locations, in which an OTU was found) plotted against log10-transformed accumulated read counts (= sum of all observations in all occupied sites), colored by their ratio (Counts/Sites; non-log!). The OTU table was rarefied to even depths before plotting. Note that the occupancy was not checked for site connectivity, so no abundance/area relation can be assumed. Modified with permission from Regan et al. (2014).

FIGURE S2 **(A,B)**: Principal Coordinate Analysis (PCoA) ordination of weighted **(A)** and unweighted **(B)** UniFrac distances on a rarefied dataset on three axes.. The emperor plot is turned to best reveal the nine irregular sites found in June. **(C)**: NMDS of Bray Curtis distances after the dataset was scaled to total sample sums. The insert in the top-right corner exemplarily shows the clustering of April and May samples.

FIGURE S3 Local contribution to overall β-diversity (LCBD). Each panel represents the sampling grid in each sampling month (see Supplementary Figure S1). Each circle represents one sampling location/site. Circle size indicates LCBD value. Red circles indicate a statistically significant LCBD (*P* < 0.05) after adjustment for multiple tests.

FIGURE S4 Visualization of Table 1. Temporal and spatial variability of α- and β-diversity measures in the SCALEMIC plot. Depicted are means and standard deviations of the local contribution to β-diversity (LCBD), derived from Bray-Curtis dissimilarities of the total sum scaled OTU table species richness (Hill number 0, ^0^D), and of the linearized Simpson diversity (Hill number 2, ^2^D). Shared letters represent groups of statistically insignificant pairwise comparisons (contrasts of estimated marginal means (EMM) on generalized least squares models (GLS) after correction for spatial autocorrelation).

FIGURE S5 Abundance-independent β-diversity, expressed as multiple site dissimilarities using the S rensen-Index, partitioned into components of turnover and nestedness (Baselga, 2010). The indices were calculated for increasing number of OTUs after sorting by decreasing site frequency. 1398 OTUs were present in all samples and composed a subset of resident OTUs, for which the species turnover must be zero (β_Sør_ = 0). Labels inside the plot indicate the total relative abundance, which was represented by the subset. “1” marks the result of the total community.

FIGURE S6 Changes in relative abundances on phylum level. Annotated by RDP-Classifier on all reads (before OTU clustering). Count data scaled to all bacterial observations. Red lines indicate the mean at each sampling date. Shared letters represent groups of statistically insignificant pairwise comparisons (contrasts of estimated marginal means (EMM) on generalized least squares models (GLS) after correction for spatial autocorrelation).

FIGURE S7 Changes in relative abundances for the 32 most abundant sub-phylum groups (mostly class level), as annotated by RDP-Classifier on all reads (before OTU clustering). Count data scaled to all bacterial observations. Labels and colorization according to Supplementary Figure S6.

FIGURE S8 Relative abundances of the 20 most abundant OTUs (total fraction of all bacterial reads > 0.1%, in all cases). Annotation to the last known rank ((g) = genus, (f) = family). Count data scaled to all bacterial observations. Black lines follow the mean at each sampling date. Shared letters represent groups of statistically insignificant pairwise comparisons (see Supplementary Figure S7).

FIGURE S9 Partial additive functions of OTUs, which were differentially active in irregular sites and their environment interactions. In each panel, the partial response functions of an OTUs to a given environmental parameter are overlaid. The y-coordinate should be interpreted as the effect of the partial function after adjusting for all other variables (on log(y), with y being the absolute read count of the OTU, related to total bacterial read counts), centered around zero. Note the individual scales of each panel. UAF = Unassigned family within the last known taxon.

FIGURE S10 Partial additive functions of selected bacteria-environment interactions. In each panel, the partial response functions of single OTUs to a given environmental parameter are overlaid. The y-coordinate should be interpreted as the effect of the partial function after adjusting for all other variables (on log(y), with y being the absolute read count of the OTU, related to total bacterial read counts), centered around zero.

TABLE S1 Taxonomic affiliation of the 22 most abundant OTUs in the SCALEMIC dataset (SILVA NR 128). Each OTU represents at least 0.1% of all reads across all samples. RA = Relative abundance accumulated over the entire data set (all assigned reads/all eubacterial reads ^∗^ 100 = %).

TABLE S2 Distance based redundancy analysis (using Bray Curtis dissimilarities) of the full OTU table and environmental variables after forward selection with a double stop criterion. The model was statistically significant (F_(14,343)_ = 8.41, *P* = 0.001, adjusted R^2^ = 22.5). *P* values for model terms were obtained with function anova.cca (by = “term”, package ‘vegan’). DF = degrees of freedom. Significance codes: 0 ^∗∗∗^ 0.001 ^∗∗^ 0.01 ^∗^ 0.05.

TABLE S3 Variable selection with boosted GAMs per taxon. Values (except for ‘Total OTUs’) are given in % and should be read as the percentage of OTU per taxon for which a variable was selected. Results are shown after cross-validation (sheet 1) and after additional stability selection of the models (sheet 2). Colored symbols reflects selection frequencies (red to black = decreasing selection frequency). Abbreviations: C__*mic*_/N__*mic*_ = Microbially bound carbon/nitrogen, EOC/EON = Extractable organic carbon/nitrogen, SM = Soil moisture, SOC = Soil Organic carbon. Fungi/Protozoa/PLFA.T = PLFA abundances of Fungi/Protozoa/all other organisms.

TABLE S4 Mantel correlation coefficients between environmental variables and spatial distances (Spearman rank correlation, 999 permutations, FDR adjusted *P* values < 0.05). Blank cells indicate non-significant correlations. Absent environmental variables (c.f. Supplementary Figure A.2) did not show spatial dependency at any time point.
