# Supplementary material for: Stochastic Dispersal Rather Than Deterministic Selection Explains the Spatio-Temporal Distribution of Soil Bacteria in a Temperate Grassland
Source: Front Microbiol. 2020 Jun 30;11:1391. doi: 10.3389/fmicb.2020.01391 (PMC7338559; doi:10.3389/fmicb.2020.01391)
Supplement: FIGURE S1 — Log10 transformed frequencies (sampling locations, in which an OTU was found) plotted against log10-transformed accumulated read counts (= sum of all observations in all occupied sites), colored by their ratio (Counts/Sites; non-log!). The OTU table was rarefied to even depths before plotting. Note that the occupancy was not checked for site connectivity, so no abundance/area relation can be assumed. Modified with permission from Regan et al. (2014). [file Data_Sheet_1.docx]

**Supplement to**

**"Stochastic dispersal rather than deterministic selection explains the spatio-temporal distribution of soil bacteria in a temperate grassland"**

Richter-Heitmann, Tim; Hofner, Benjamin; Krah, Franz-Sebastian; Sikorski, Johannes; Wüst, Pia K.; Bunk, Boyke; Huang, Sixing; Regan, Kathleen M.; Berner, Doreen; Boeddinghaus, Runa S.; Marhan, Sven; Prati, Daniel; Kandeler, Ellen; Overmann, Jörg, and Michael W. Friedrich

## Glossary of frequently used terms and expressions

| **Term** | **Explanation** |
| --- | --- |
| Abundance | Throughout the paper, the term "abundance" refers to the amount of rRNA sequence reads of a given OTU in a sample or dataset. |
| Autocorrelation | The degree of dependency among observations in space or time. May depend on external effects (e.g. water availability) or represent its own process. In *P* value based inferential statistics, autocorrelation violates the assumption of independent measurements. Numerous ways to deal with autocorrelation exist (e.g., permutation or using correlation structure arguments). |
| Boosting | Statistical algorithm to fit linear or generalized additive models (🡪 GAMs) and to select important variables (🡪variable selection) at the same time. |
| C_mic_,N_mic_ | The fraction of soil organic carbon/nitrogen, which is defined as microbial biomass. |
| Cross-validation | Statistical algorithm to validate model performance on “new data” by repeatedly splitting the data into two parts, one to fit the model and one to evaluate the model; by this approach one tries to assess how good the results can be generalized or if they are specific to the data at hand (overfitting). |
| Deterministic processes | Processes that shape communities under conditions in which competing species are not equally adapted to the habitat. Synonymous to e.g., environmental selection (or filtering), or niche-based processes. Selection can act as hetero- (strong environmental gradients) or homogenizing effect (identical environmental conditions along a spatial gradient, which still act as filters). |
| Dispersal | Movement of species/propagules in space and time. If selective pressure is low or absent, dispersal is conceptually neutral, but dispersal can depend on group specific traits, as well. However, Microbial dispersal is largely viewed as stochastic (Zhou and Ning, 2017). If dispersal is limited, communities are unlinked, and migration is stopped. |
| Drift | 🡪 Stochastic changes of species abundances, e.g., by oscillating generation times (random deaths/births). Most important if 🡪 selection is weak. |
| Functional redundancy | The ability of a community to have multiple species provide the same ecosystem service, as a result of evolutionary convergence. High functional redundancy increases the prevalence of stochastic assembly processes. |
| EOC, EON | Extractable organic carbon or nitrogen: The fraction of the soil organic matter (SOM), which is associated with bio-available organic matter in bigger soil pores (Chantigny, 2003), and extractable with water or aqueous solutions of some salts, such as K_2_SO_4_ or CaCl_2_. |
| Frequency | The number of observed sampling stations in which a given OTU was observed. |
| GAM (generalized additive model) | A modelling framework in which the linear covariates of a basic linear model Y = b_0_ + b_1_x_1_ + b_2_x_2_+... +b_n_x_n_ can be replaced with functions: Y = b_0_ + f_1_(x_1_) + f_2_(x_2_) +... + f_n_(x_n_). Functions f_i_ may be specified by parametric, non- and semi-parametric forms (🡪 smoother). Thus, Y is no longer a linear combination of covariates (but additive in the functions). |
| GAMLSS (generalized additive model for location, scale and shape) | GAMLSS are an extension of generalized additive models (🡪 GAM) that allow all parameters of a distribution (such as mean, variance, or skewness) to be modeled as a linear or additive (🡪 GAM) function of covariates. Here, GAMLSS are used to fit zero-inflated negative binomial models, where the mean is modeled using an additive model, while overdispersion and zero-inflation (the other two parameters of the distribution) are treated as constants, i.e., might be present but are not allowed to depend on covariates. |
| Neutral processes | Processes that shape communities under conditions in which  competing species are equally adapted to the habitat.  🡪 Stochasticity is often used synonymously, although the assumption of equal adaptation is not necessarily made. |
| βNTI | β-Nearest Taxon Index: the phylogenetic turnover between two communities. Calculation is based on the βMNTD (Mean Nearest Taxon Distance). The βNTI is hereby defined as  βNTI = $\frac{{\beta MNTD}_{\mathrm{Observed}}- \overline{\beta MNTD}_{Null}}{sd ({\beta MNTD}_{Null})}$  βMNTD_Null_ is derived from randomizing the two communities in comparison (🡪 Null models). Repeating the process yields mean and standard deviation of βMNTD_Null_, which can be compared to the observed βMNTD to indicate if the observed phylogenetic distance is lesser, higher or equal than expected by random chance alone. The higher the deviation of βNTI from zero, the higher is the likelihood of environmental 🡪 selection. Both indices exist as within-sample versions (NTI/MNTD). In the framework of Stegen et al., 2013, the βNTI separates neutral from deterministic community assembly. |
| Null models | "A pattern-generating model that is based on randomization of ecological data" (Gotelli and Graves, 1996). The generated random pattern represents changes by chance alone (but see (Tucker et al., 2016), in the absence of ecological mechanisms. Often realized as random draws from a meta-community (i.e. all observed species in the experimental context). In this study, null models were used for 🡪 βNTI generation and 🡪Raup-Crick dissimilarities. |
| Plot scale | A single site with homogenous properties (vegetation, topography, climate, and management history), spanning up to not more than several hundred meters in both directions. |
| Raup-Crick (dissimilarities) | A metric, which quantifies the ratio of the number of shared species between two sites to the number of shared species by random chance, based on the species pool in the entire data set and in the two compared sites. This traditionally abundance-unweighted metric was extended to weighted abundances in Stegen et al., 2013. This approach is also used in this study. |
| Residents | OTUs with high site 🡪 frequency, i.e. a cosmopolitan lifestyle in regards to the investigated site (🡪 Transients). |
| Richness | A standardized measure of α-diversity, e.g. the number of phylotypes or species. In this study, we use two richness parameters, the unweighted, total species richness (*^0^D*) and a linearized version of the Simpson Diversity (*^2^D*), which gives more weight to dominant species. The terms *^0^D* and *^2^D* indicate their status as Hill numbers (Chao et al., 2014). |
| Stability | The tendency of communities to return to a pre-disturbance or alternative stable state after being subjected to in- or external disturbances. |
| Selection | Processes leading to the prevalence of populations which are better adapted to current environmental conditions. Species sorting, environmental filtering and niche effects are frequently used terms referring to the same ecological process. |
| Smoother | Algorithm to fit functions f_1_(x_1_), …, f_p_(x_p_) to the data such that one obtains a smooth curve through the data without big jumps (🡪 GAMs); sometimes also refers to the resulting function estimate. |
| Stochasticity | Random changes in community structure in respect to species identities and abundances due to stochastic processes of birth, death, migration, spatiotemporal variation and historical contingency, as recently defined by Zhou and Ning (2017). Examples are 🡪 stochastic dispersal, 🡪drift or speciation/diversification (not considered here). See also 🡪 Neutral processes. |
| Transients | OTUs with low site 🡪 frequency, usually part of the rare biosphere. |
| Variable selection | A) Mathematical/statistical algorithms to identify the meaningful predictors in a regression model to obtain parsimonious models  B) In the framework of Stegen et al. (2013, 2015), variable selection refers to 🡪 selection causing heterogeneous species sorting. |
| Variance partitioning | A method to identify those fractions of the variation in a dataset, which are explained by specified predictor categories (e.g. space and environment), either as pure or joint fractions. |

## Section A: Experimental design and environmental data

#
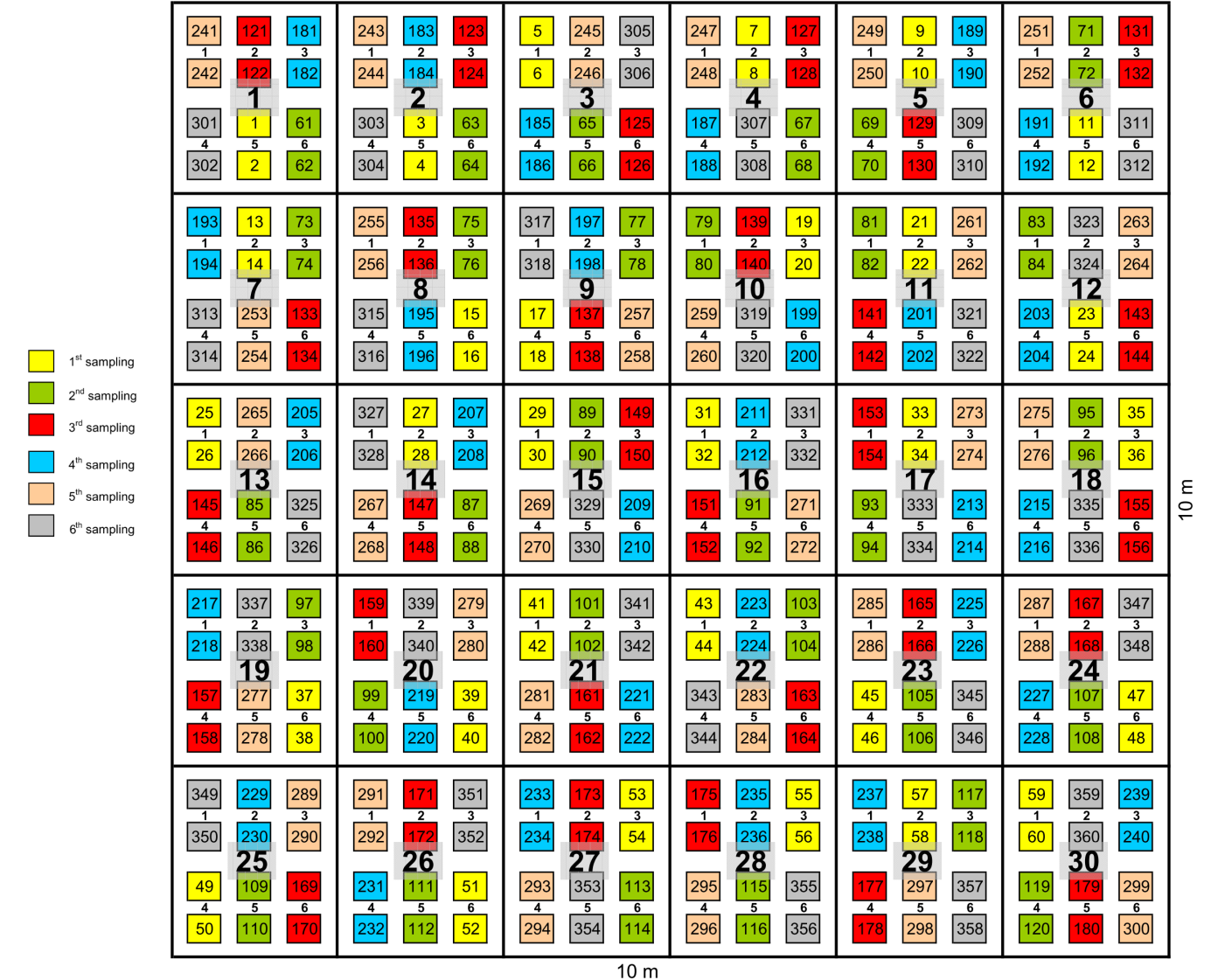


A.1: The SCALEMIC sampling design (modified after Regan et al. (2014)). The scheme describes a square of 100 m^2^. This plot was divided into 30 subplots. Each subplot was sampled twice in two neighboring locations during each sampling location, at a distance of 50 cm between two neighbors.


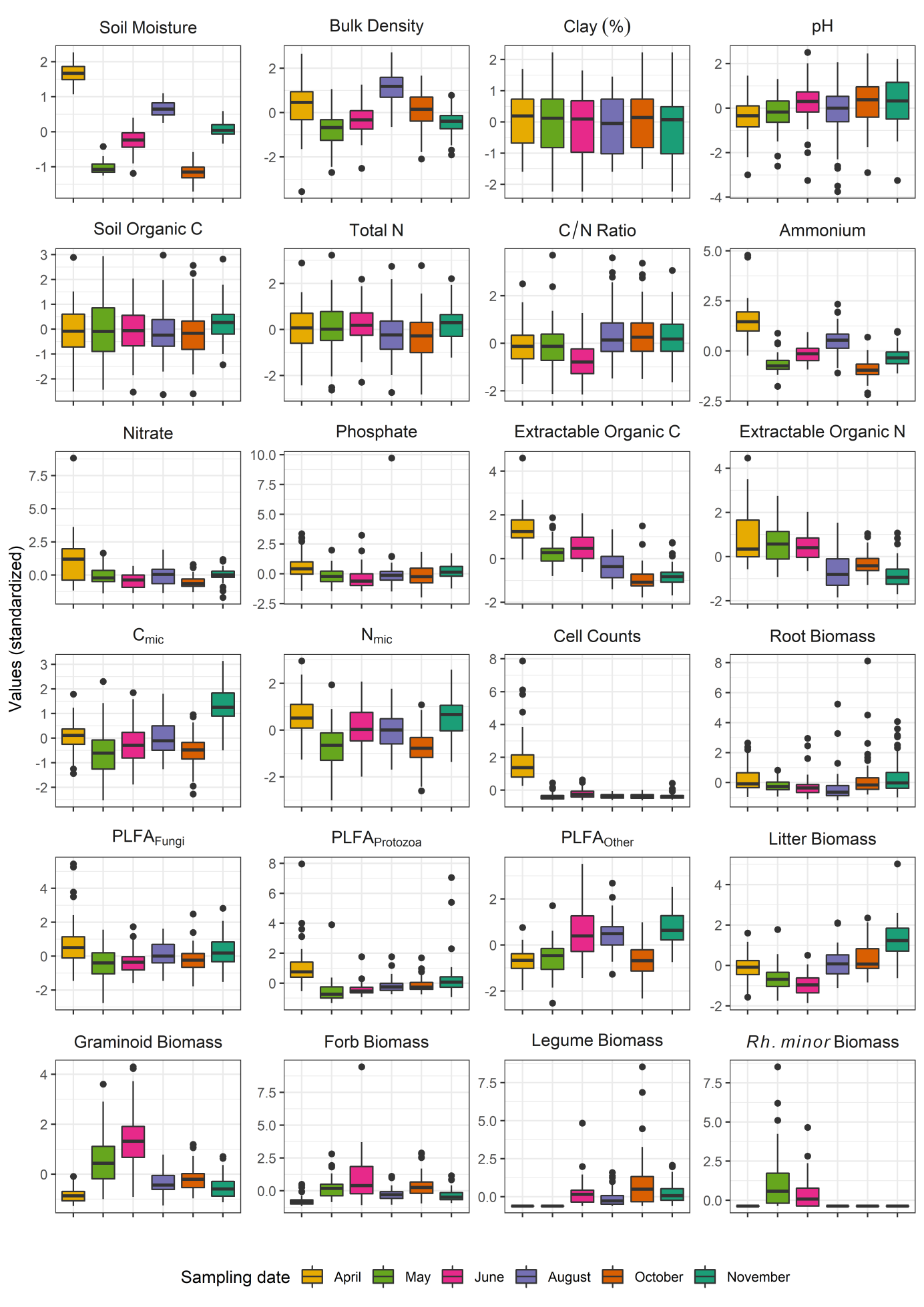


A.2: Boxplots depicting the seasonal progression of environmental variables in the experimental site. Values are standardized (mean = 0, SD = 1). Two PLFA (phospholipid-derived fatty acids) signals were used to represent saprophobic fungi (18:2ω6) and invertebrates (20:4ω6), the remaining PLFA signals were combined into a single variable ("PLFA_Other_", as a proxy for microbial productivity). C_mic_, N_mic_ = Microbially bound carbon/nitrogen. Cell counts refer to prokaryotic cells. For experimental procedures, unscaled values, and *post hoc* analysis, see Regan et al. (2014).


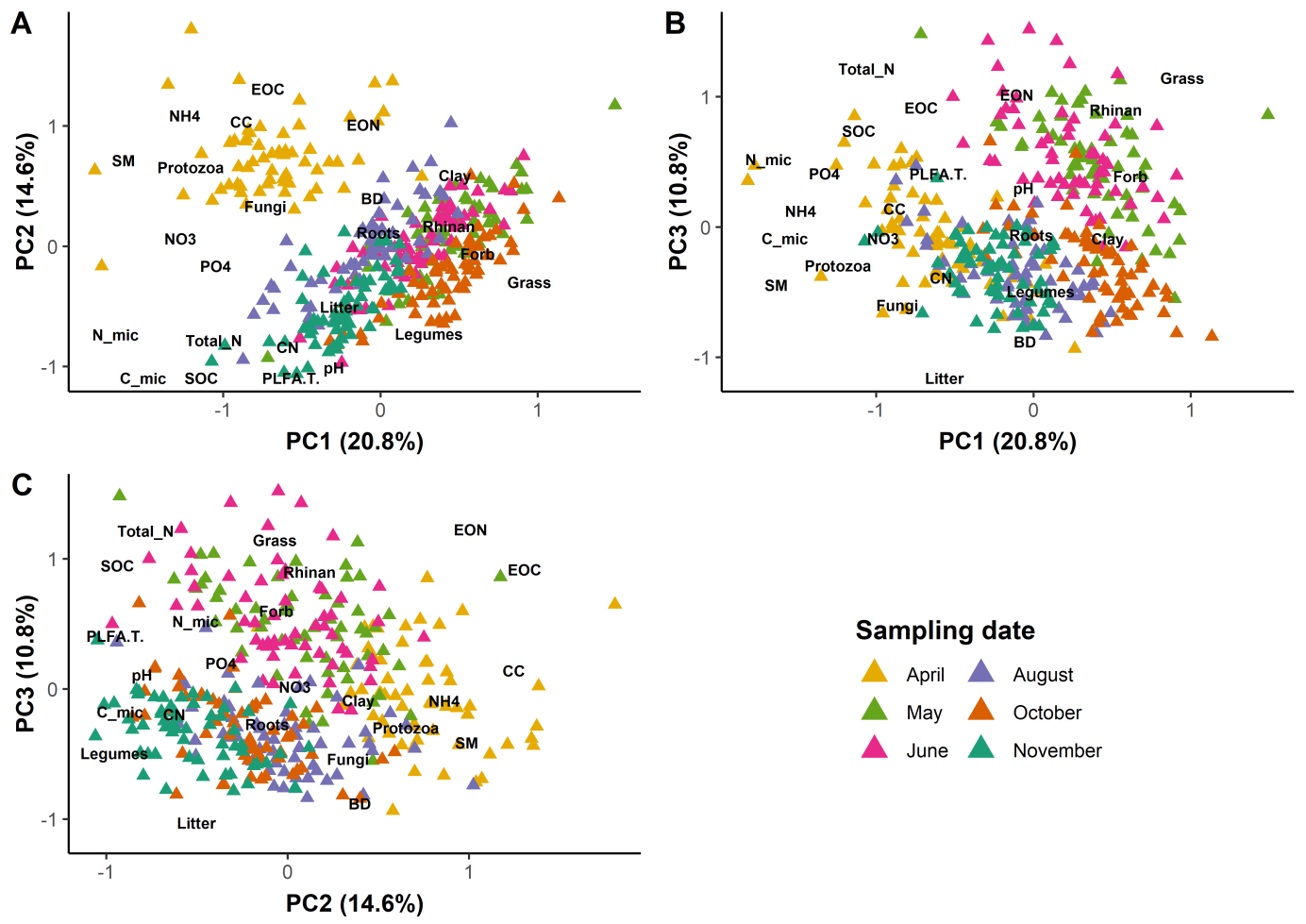


A.3: Principal Component Analysis (PCA) of 24 environmental variables taken from the SCALEMIC site, colored by sampling date. Values are standardized (mean = 0, SD = 1). Three PCA axes were significant as per broken stick analysis (PC1 – 3). Biplot ordination of sites and environmental variables; arrows were omitted, but labels are positioned at their projected tip coordinates. The first two axes separate April from all other samples based on various. Axis 1 and 3 separate May and June from other samples based on plant biomass gradients. Abbreviations: BD = Bulk density, CC = Bacterial cell counts, C_mic/N_mic = Microbially bound carbon/nitrogen, EOC/EON = Extractable organic carbon/nitrogen, SM = Soil moisture, SOC = Soil Organic carbon. Fungi/Protozoa/PLFA.T = PLFA abundances of Fungi/Protozoa/all other organisms.

## Section B: Preliminary analysis

From the initial 597 million raw eubacterial reads, 455 million reads could be annotated as Bacteria by the RDP-Classifier, after trimming and quality filtering. 102.5 million reads could be clustered into 16,944 reference OTUs after removal of global single- and doubletons, chloroplasts and mitochondria. In this dataset, the progression of the Simpson diversity index during rarefaction demonstrated that the abundant OTUs were completely captured after subsampling of about 50,000 reads for each sample, while the raw species richness approximated saturation at the targeted reference sample size (see Figure B.2).


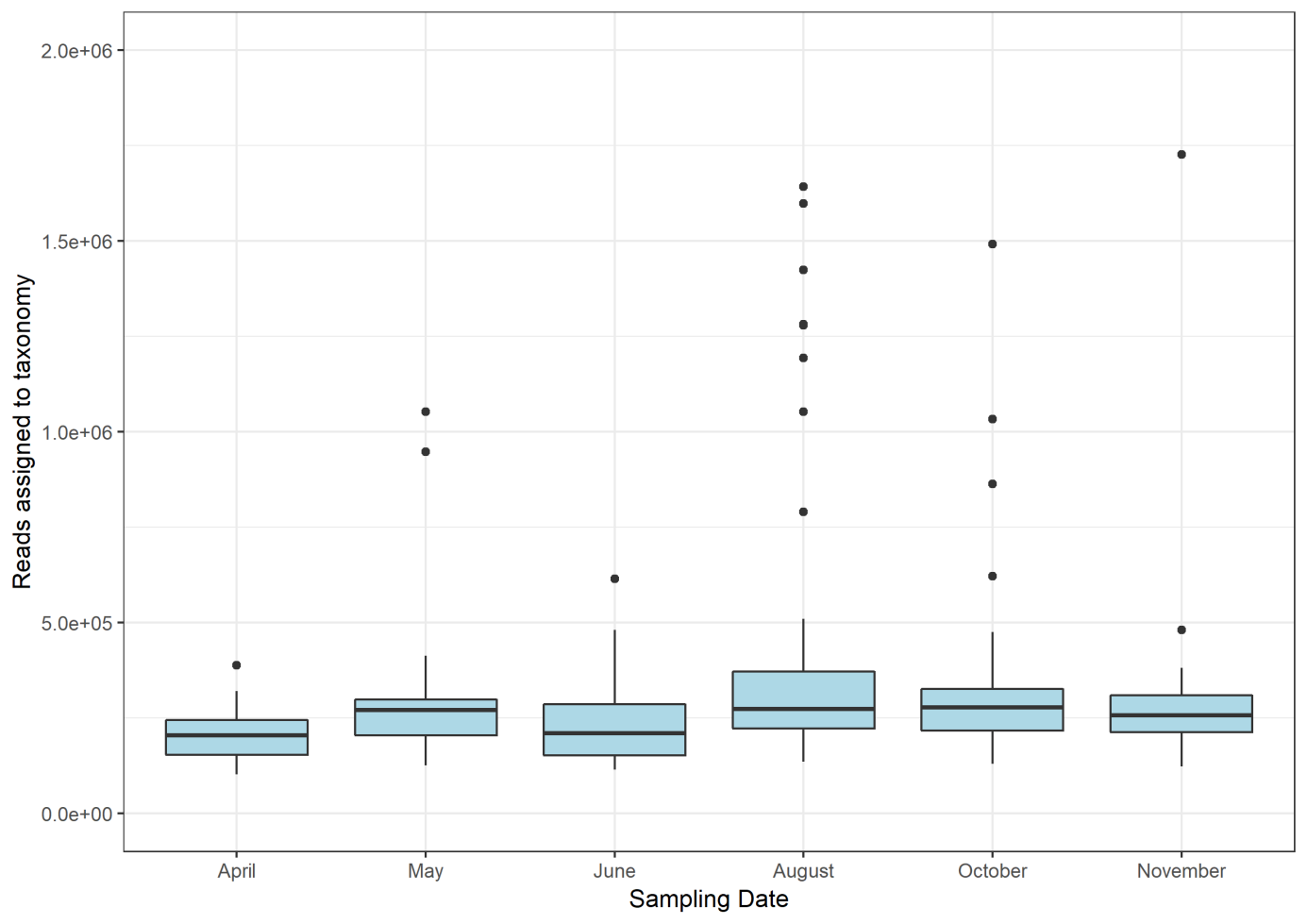


B.1: Illumina HiSeq II reads per sample, sorted by sampling date. Shown are reads assigned to bacterial OTUs included in the reference database (SILVA Ref NR 128).


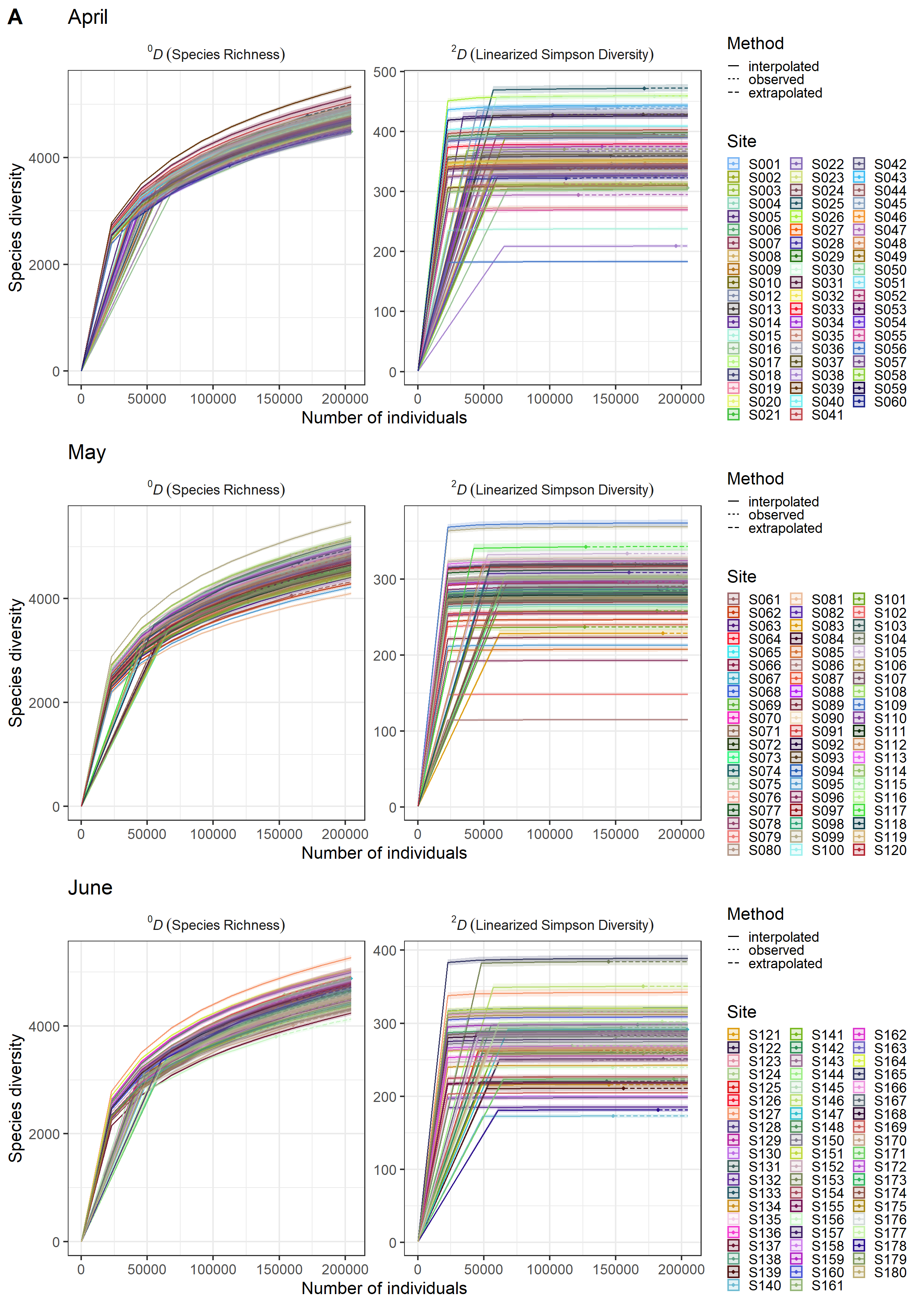


**B.2: Rarefaction analysis sorted by sampling date. A: April to July 2011. Dependent on unrarefied sample size, OTU counts were inter- or extrapolated to twice the smallest sampling size, according to Chao et al., 2014. We calculated species richness (*^0^D*) and a linearized form of the Simpson diversity index (*^2^D*). B (next page): August to November 2011.**

**
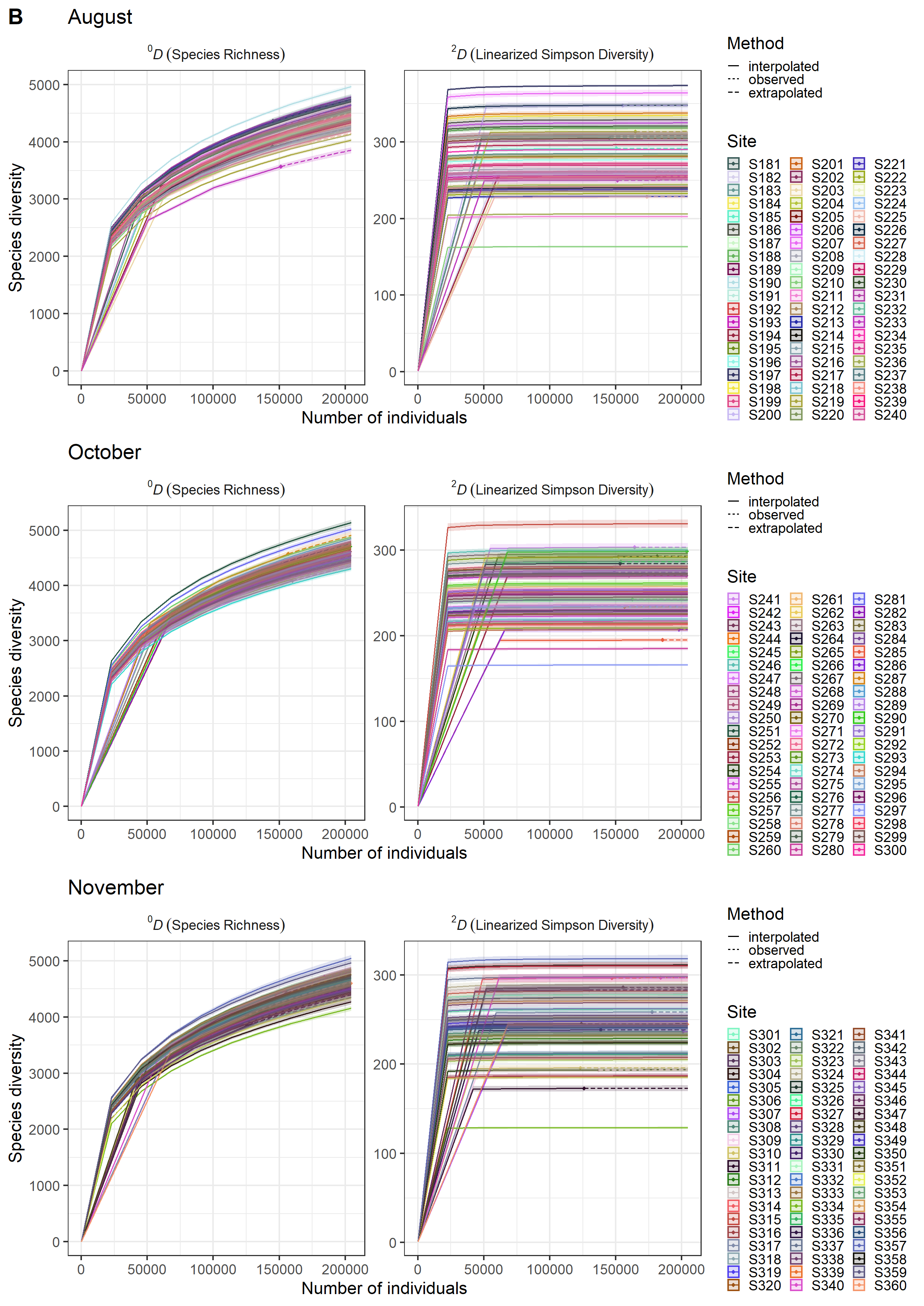
**

**B.2 (cont.): Rarefaction analysis sorted by sampling date. B: August to November 2011. Dependent on unrarefied sample size, OTU counts were inter- or extrapolated to twice the smallest sampling size, according to Chao et al., 2014. We calculated species richness (*^0^D*) and a linearized form of the Simpson diversity index (*^2^D*).**

## Section C: *Post Hoc* analysis

During data analysis, the need for a reliable *post hoc* test approach was identified. Realizing that different methods yielded massively different results, we finally opted for a consensus approach (Extended Figure 3). Thus, differential abundance of OTUs between specific groups of sites was assessed utilizing the following methods:

1. functions in package ANCOM (Mandal et al., 2015)
2. estimation of marginal means as described in the main text (Lenth, 2018)
3. the general linear hypothesis test in package 'multcomp' (Hothorn et al., 2008; Herberich et al., 2010)
4. functions in package DESeq (Love et al., 2014)
5. functions in package edgeR (Robinson et al., 2010),
6. Welch’s t-test as implemented in STAMP (Parks et al., 2014).

Significant differences in abundance were affirmed if any five of the six tests agreed on outcome. If not automatically adjusted, p values were corrected for multiple testing with the 'FDR' method (Benjamini and Hochberg, 1995). ANCOM (Mandal et al., 2015) was developed to control false discovery rates due to potential compositional effects in the data. We note that ANCOM – while being the most conservative *post hoc* test in our results – did not yield any unique results, and was largely in agreement with conventional ANOVA-type post-hoc tests on relative count data (e.g., general linear hypothesis test, Welch’s t-test).


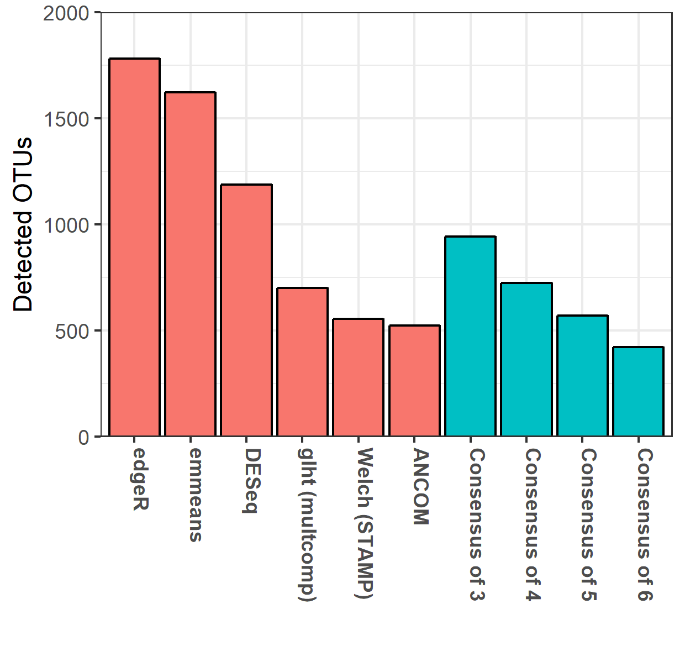


**C.1: Comparison of differential abundance tests (red bars) and consensus results between any *n* tests (turquois bars). The y-axis represents the number of OTUs, which were differently abundant between regular and irregular sites in June.**

## Section D: Parallelization of abundance-based Raup-Crick dissimilarity assessments





**D.1: Benchmark comparison between a parallelized (this study) and a non-parallelized script to estimate abundance-weighted Raup-Crick distances (Stegen, et al., 2013). A real Illumina sequencing data set was subdivided by constant OTU numbers vs. a sample gradient (left panel) and vice versa (right panel). Scripts were run under 999 permutations on six CPU cores.**

## Section E: Variable selection with boosted generalized additive models

Generalized additive models (GAMs) and generalized additive models for location, scale and shape (GAMLSS) were fitted to individual OTUs separately to model their abundances. We used negative binomial models (i.e., GAMs) for cosmopolitan species and zero-inflated negative binomial models (i.e., GAMLSS) for species with higher endemicities (> 40 plots with zero observations). In the latter case, we only modeled the mean as a function of covariates, while treating overdispersion and zero-inflation as constants. During model fitting, non-normalized counts were used for each OTU. However, we specified the (logarithm of) total bacterial counts per plot as offset to implicitly model relative counts. This offset was used as a first step in the boosting algorithm.

The models accounted for environmental, temporal and spatial effects. Categorical effects were used to model the temporal structure, P-splines (Eilers and Marx, 1996; Schmid and Hothorn, 2008) were used to model smooth, environmental effects, and bivariate P-splines were applied for spatial effects (Kneib et al., 2009). In a second step, spatio-temporal effects were added to the model to account for possible changes over time. These effects were modeled as time-specific bivariate P-splines. The derived effects can be interpreted in the same way as classic maximum likelihood estimates, while the resulting model is estimated in a sequential fashion such that an optimal prediction accuracy is obtained (i.e., such that the outcome is optimally predicted for new data).

In order to fit the models, we used component-wise boosting methods (see Mayr et al. (2014) for an overview): Each effect is specified as a so-called base-learner. Each of the base-learners is then fitted separately to the negative gradient of the loss function evaluated at the current fit (here the positive gradient of the log-likelihood) and only the best-fitting effect is updated. This is done by adding a fraction of the fit (usually 10%) to the model and by updating the corresponding effect estimate accordingly. In the next step, the negative gradient is recomputed and the procedure is iterated until a pre-specified number of iterations is reached. For details on the algorithm, refer to Hofner et al. (2014).

This number of boosting iterations is the major tuning parameter of the model. By using cross-validation methods (such as 10-fold cross-validation, bootstraps or subsampling), the appropriate stopping iteration is obtained such that the prediction accuracy of the model is optimized for new data (Mayr et al., 2012). Here, we used 25-fold bootstrap to obtain the optimal stopping iteration for each model. As only one base-learner is updated per iteration, we obtain variable selection by stopping the model after a sufficiently small number of steps (“early stopping”). Hence, boosting with cross-validation allows to fit the model and to select variables at the same time. The resulting model is usually sparse and has good prediction performance.

Yet, in many situations, boosting is known to select too many noise variables (i.e., variables that in reality do not have an influence on the outcome). To obtain even sparser models, we additionally applied the generic stability selection approach (Meinshausen and Bühlmann, 2010; Shah and Samworth, 2013). Stability selection uses 100 random subsamples of size ⌊n/2⌋. The model is fitted on each of the subsamples until *q* variables have been selected by the boosting algorithm. Variables that were selected on each (or at least most) of the subsamples are considered as stable, i.e., important effects. To determine how often a variable has to be selected to be stable one uses a threshold for the selection frequency. This threshold can in turn be computed from a pre-specified bound on the per-family error rate (PFER). The PFER is defined as the expected number of falsely selected variables, i.e., as the expected number of noise variables that were selected. We specified *q* = 12 and PFER ≤ 2, i.e., we accepted at most two noise variables to be selected in each model which resulted in a threshold for the selection frequency of 80%. For details, see Hofner et al. (2015).

## 2. Supplementary Tables

**Supplementary Table 1. Taxonomic affiliation of the 22 most abundant OTUs in the SCALEMIC dataset (SILVA NR 128). Each OTU represents at least 0.1% of all reads across all samples. RA = Relative abundance accumulated over the entire data set (all assigned reads / all eubacterial reads * 100 = %).**

| RA | OTU-ID | Phylum | Class | Order | Family | Genus |
| --- | --- | --- | --- | --- | --- | --- |
| 0.606 | 14838 | Proteobacteria | Alphaproteobacteria | Rhizobiales | Bradyrhizobiaceae | Bradyrhizobium |
| 0.458 | 16077 | Proteobacteria | Alphaproteobacteria | Rhizobiales | Xanthobacteraceae | Variibacter |
| 0.348 | 5009 | Actinobacteria | Actinobacteria | Propionibacteriales | Propionibacteriaceae | Microlunatus |
| 0.301 | 22882 | Verrucomicrobia | Spartobacteria | Chthoniobacterales | Xiphinematobacteraceae | Cand. Xiphinematobacter |
| 0.251 | 17028 | Proteobacteria | Alphaproteobacteria | Rhodospirillales | - | Cand. Alysiosphaera |
| 0.207 | 346 | Acidobacteria | Blastocatellia | Blastocatellales | Blastocatellaceae | 11-24 |
| 0.200 | 15277 | Proteobacteria | Alphaproteobacteria | Rhizobiales | Hyphomicrobiaceae | Rhodoplanes |
| 0.193 | 1645 | Acidobacteria | SG6 | - | - | - |
| 0.183 | 4761 | Actinobacteria | Actinobacteria | Propionibacteriales | Nocardioidaceae | Kribbella |
| 0.148 | 5197 | Actinobacteria | Actinobacteria | Pseudonocardiales | Pseudonocardiaceae | Pseudonocardia |
| 0.148 | 5857 | Actinobacteria | MB-A2-108 | - | - | - |
| 0.145 | 22883 | Verrucomicrobia | Spartobacteria | Chthoniobacterales | Xiphinematobacteraceae | Cand. Xiphinematobacter |
| 0.141 | 5965 | Actinobacteria | Rubrobacteria | Rubrobacterales | Rubrobacteriaceae | Rubrobacter |
| 0.139 | 15940 | Proteobacteria | Alphaproteobacteria | Rhizobiales | Rhodobiaceae | - |
| 0.133 | 5417 | Actinobacteria | Actinobacteria | Streptomycetales | Streptomycetaceae | Streptomyces |
| 0.130 | 17042 | Proteobacteria | Alphaproteobacteria | Rhodospirillales | - | Reyranella |
| 0.129 | 1975 | Acidobacteria | SG6 | - | - | - |
| 0.123 | 15540 | Proteobacteria | Alphaproteobacteria | Rhizobiales | Phyllobacteriaceae | Mesorhizobium |
| 0.114 | 6025 | Actinobacteria | Thermoleophilia | Gaiellales | Gaiellaceae | Gaiella |
| 0.114 | 15541 | Proteobacteria | Alphaproteobacteria | Rhizobiales | Phyllobacteriaceae | Mesorhizobium |
| 0.113 | 4505 | Actinobacteria | Actinobacteria | Micromonosporales | Micromonosporaceae | Dactylosporangium |
| 0.109 | 13887 | Planctomycetes | Planctomycetacia | Planctomycetales | Planctomycetaceae | - |

**Supplementary Table 2. Distance based redundancy analysis (using Bray Curtis dissimilarities) of the full OTU table and environmental variables after forward selection with a double stop criterion. The model was statistically significant (F_(14,343)_ = 8.41, *P* = 0.001, adjusted *R^2^* = 22.5). *P* values for model terms were obtained with function anova.cca (by ="term", package 'vegan'). DF=degrees of freedom. Significance codes: 0 *** 0.001 ** 0.01 * 0.05**

|  | **DF** | **Variance** | **F** | ***P*** | **Significance** |
| --- | --- | --- | --- | --- | --- |
| **Soil Moisture** | 1 | 0.922 | 38.008 | 0.001 | *** |
| **pH** | 1 | 0.600 | 24.717 | 0.001 | *** |
| **Extractable Organic C** | 1 | 0.247 | 10.165 | 0.001 | *** |
| **Microbial N** | 1 | 0.235 | 9.694 | 0.001 | *** |
| **Microbial C** | 1 | 0.210 | 8.667 | 0.001 | *** |
| **Phosphate** | 1 | 0.106 | 4.362 | 0.002 | ** |
| **Soil Organic C** | 1 | 0.080 | 3.278 | 0.001 | *** |
| **Clay Content** | 1 | 0.077 | 3.165 | 0.001 | *** |
| **Litter Biomass** | 1 | 0.072 | 2.960 | 0.002 | ** |
| **Total Lipids (PLFA)** | 1 | 0.069 | 2.852 | 0.001 | *** |
| **C/N Ratio** | 1 | 0.065 | 2.697 | 0.002 | ** |
| **Graminoid Biomass** | 1 | 0.062 | 2.567 | 0.005 | ** |
| **Ammonium** | 1 | 0.059 | 2.417 | 0.005 | ** |
| **Legume Biomass** | 1 | 0.052 | 2.136 | 0.021 | * |
| **Residual** | 343 | 8.3233 |  |  |  |

**Supplementary Table 3. Variable selection with boosted GAMs per taxon. Values (except for 'Total OTUs') are given in percentage and should be read as the percentage of OTU per taxon for which a variable was selected. Results are shown after cross-validation (sheet 1) and after additional stability selection of the models (sheet 2). Colored symbols reflects selection frequencies (red to black = decreasing selection frequency). Abbreviations: C_mic/N_mic = Microbially bound carbon/nitrogen, EOC/EON = Extractable organic carbon/nitrogen, SM = Soil moisture, SOC = Soil Organic carbon. Fungi/Protozoa/PLFA.T = PLFA abundances of Fungi/Protozoa/all other organisms.**

**SEE ATTACHED EXCEL SHEET ("Supplementary Table 3.xlsx").**

**Supplementary Table 4. Mantel correlation coefficients between environmental variables and spatial distances (Spearman rank correlation, 999 permutations, FDR adjusted *P* values < 0.05). Blank cells indicate non-significant correlations. Absent environmental variables (c.f. Supplementary Figure A.2) did not show spatial dependency at any timepoint.**

|  | April | May | June | August | October | November |
| --- | --- | --- | --- | --- | --- | --- |
| **pH** | 0.14 | 0.17 |  | 0.19 |  |  |
| **Bulk Density** | 0.13 |  |  |  |  |  |
| **Soil Moisture** | 0.23 |  |  | 0.13 |  |  |
| **Clay Content** | 0.11 |  |  | 0.12 | 0.11 |  |
| **Phosphate** |  |  |  |  | 0.11 |  |
| **Soil Organic C** | 0.17 | 0.12 | 0.11 | 0.15 |  |  |
| **Total Nitrogen** | 0.17 | 0.12 |  | 0.20 |  |  |
| **C/N Ratio** | 0.13 |  | 0.13 |  |  |  |
| **Microbial C** | 0.13 |  |  |  |  |  |
| **Forb Biomass** | 0.14 |  |  |  |  |  |
| **Legume Biomass** |  |  | 0.20 | 0.14 |  |  |
| **Litter Biomass** |  |  | 0.18 |  |  |  |
| **R. Minor Biomass** |  |  | 0.15 |  |  |  |

## Supplementary Figures


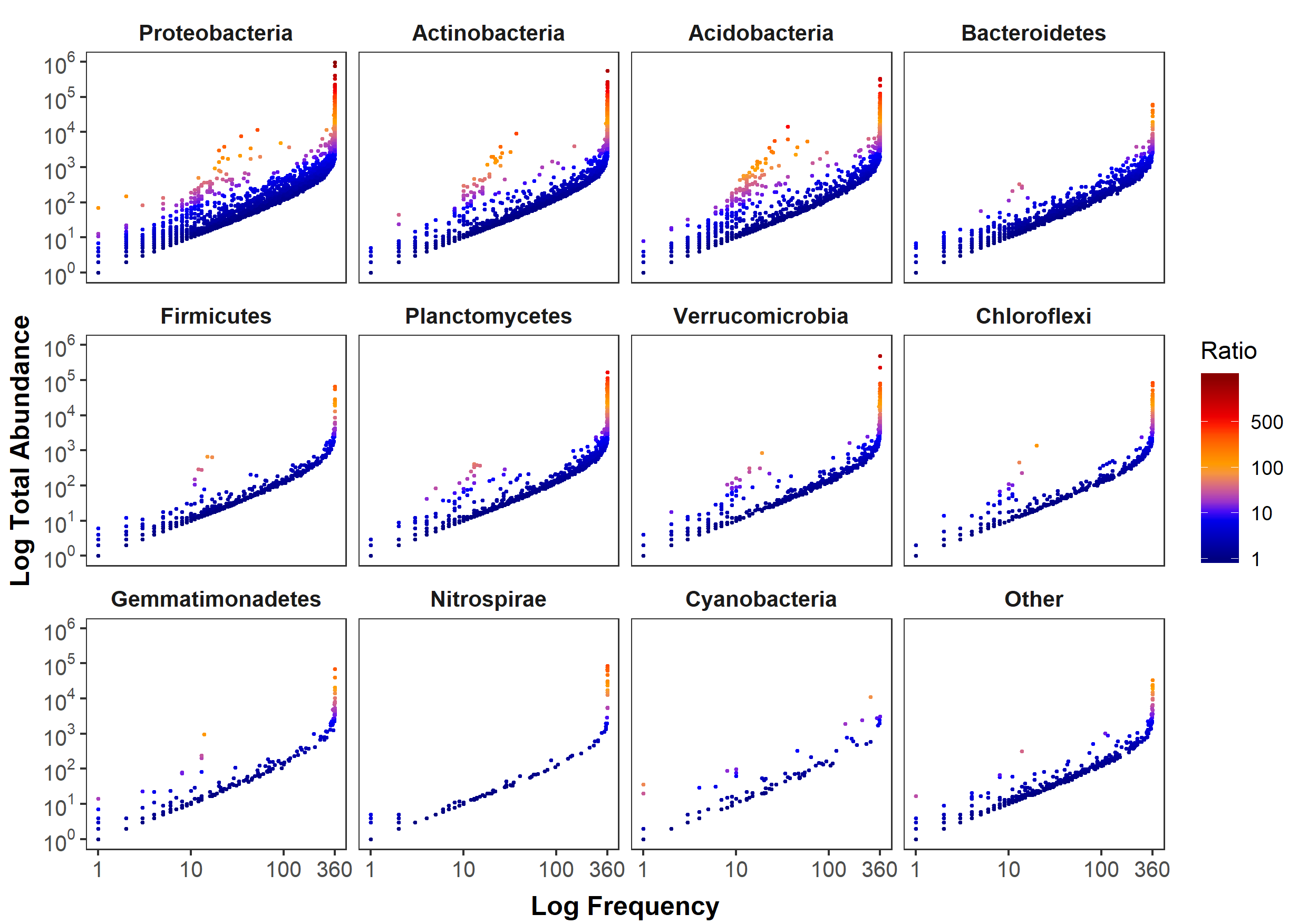


**Supplementary Figure 1. Log10 transformed frequencies (sampling locations, in which an OTU was found) plotted against log10-transformed accumulated read counts (= sum of all observations in all occupied sites), colored by their ratio (Counts/Sites; non-log!). The OTU table was rarefied to even depths before plotting. Note that the occupancy was not checked for site connectivity, so no abundance/area relation can be assumed.**


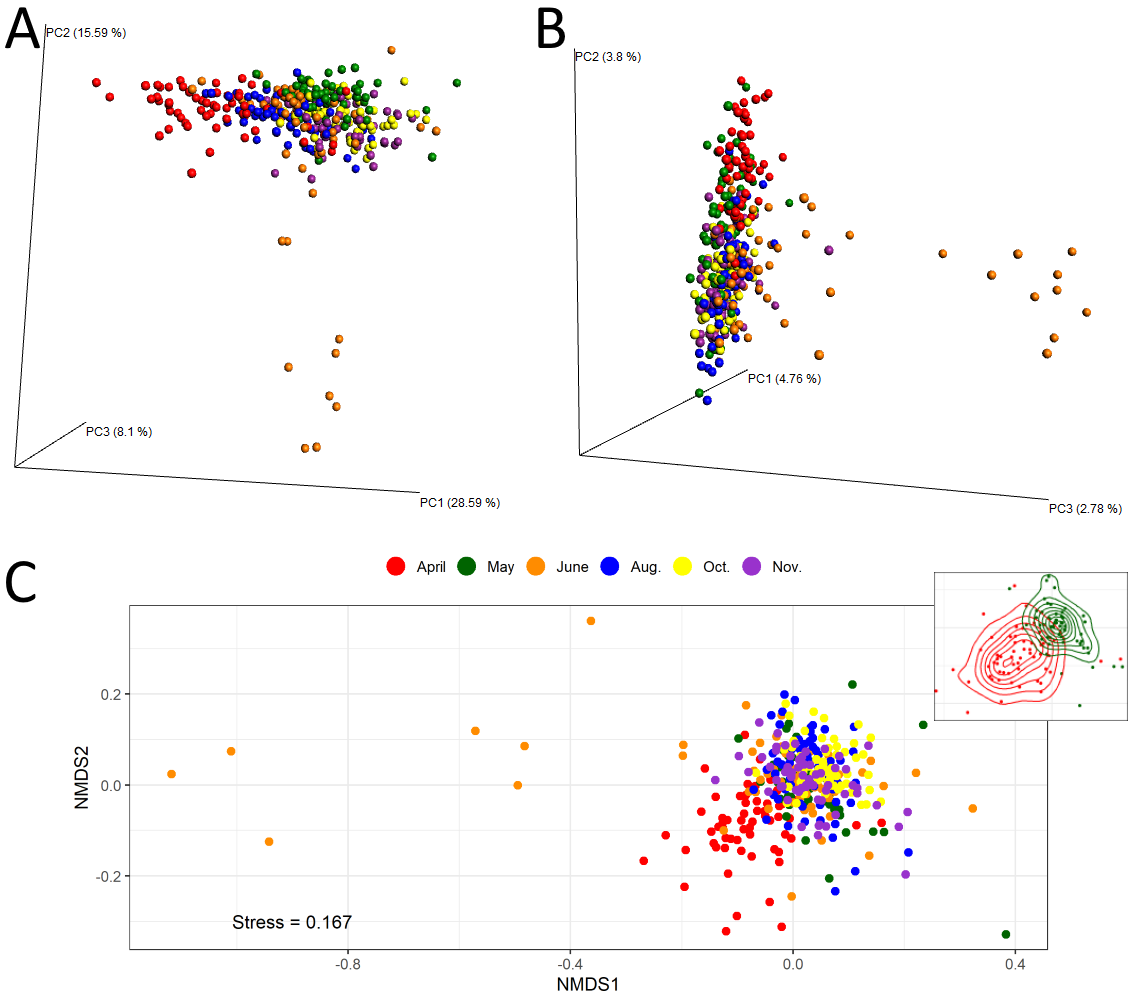


Supplementary Figure 2. A, B: Principal Coordinate Analysis (PCoA) ordination of weighted (A) and unweighted (B) UniFrac distances on a rarefied dataset on three axes. The emperor plot is visually turned to best reveal the nine irregular sites found in June. C: NMDS of Bray Curtis distances after the dataset was scaled to total sample sums. The insert in the top-right corner exemplarily shows the clustering of April and May samples.


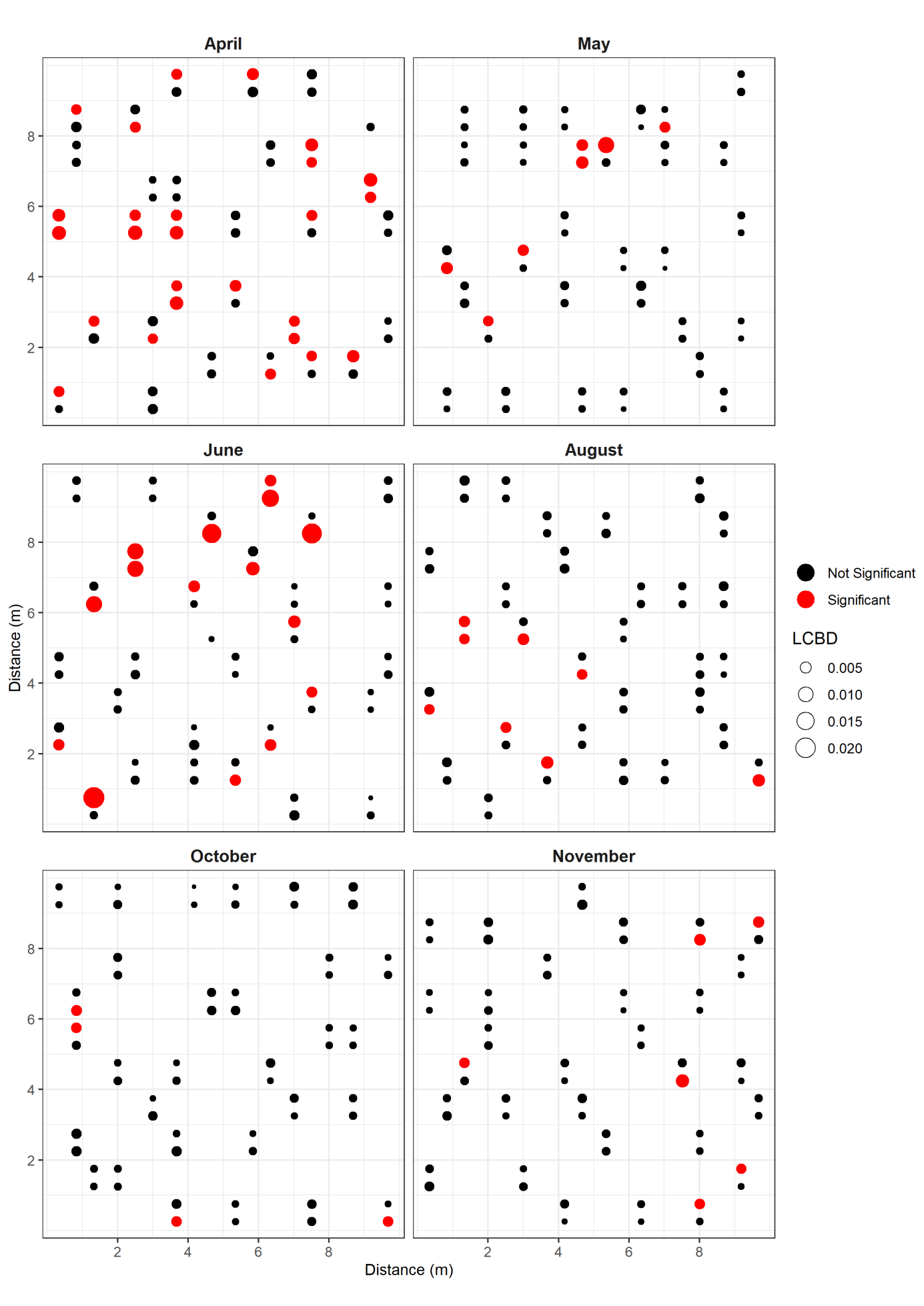


**Supplementary Figure 3. Local contribution to overall β-diversity (LCBD). Each panel represents the sampling grid in each sampling month (see Supplementary Figure 1). Each circle represents one sampling location/site. Circle size indicates LCBD value. Red circles indicate a statistically significant LCBD (*P* < 0.05) after adjustment for multiple tests.**


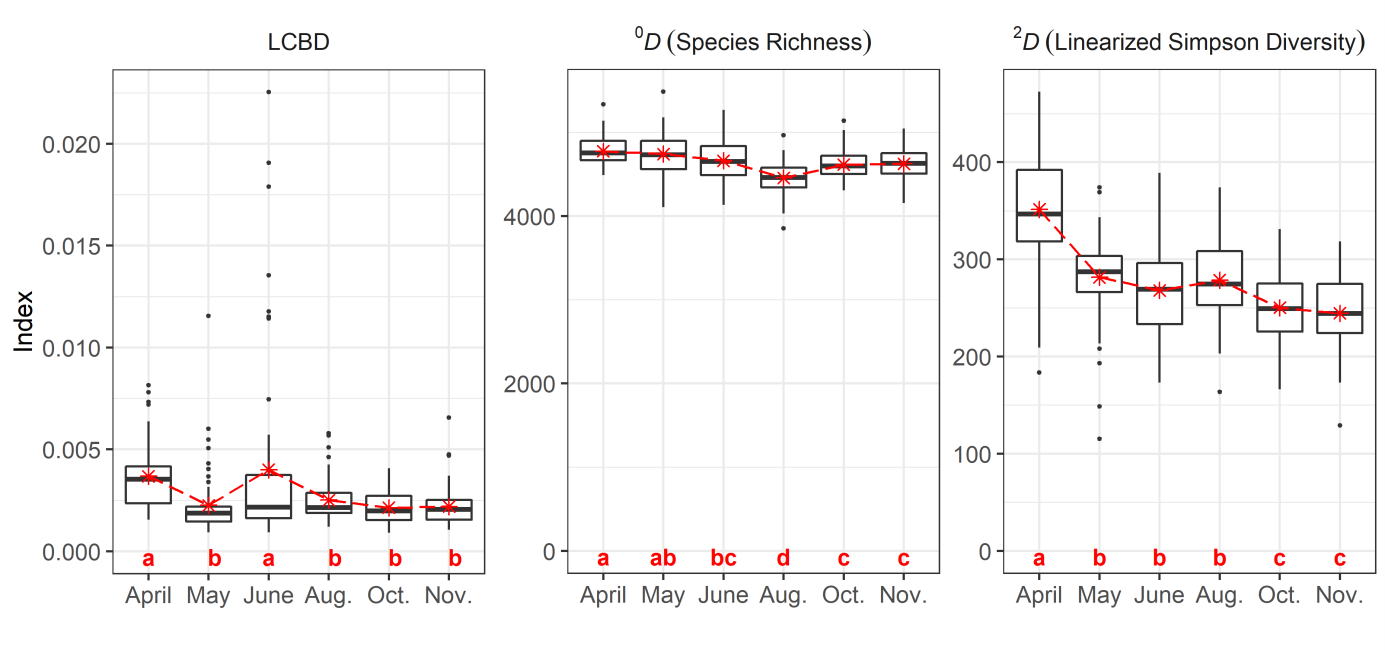


Supplementary Figure 4. Visualization of Table 1. Temporal and spatial variability of α- and β-diversity measures in the SCALEMIC plot. Depicted are means and standard deviations of the local contribution to β-diversity (LCBD), derived from Bray-Curtis dissimilarities of the total sum scaled OTU table species richness (Hill number 0, *^0^D)*, and of the linearized Simpson diversity (Hill number 2, *^2^D*). Shared letters represent groups of statistically insignificant pairwise comparisons (contrasts of estimated marginal means on generalized least squares models after correction for spatial autocorrelation).


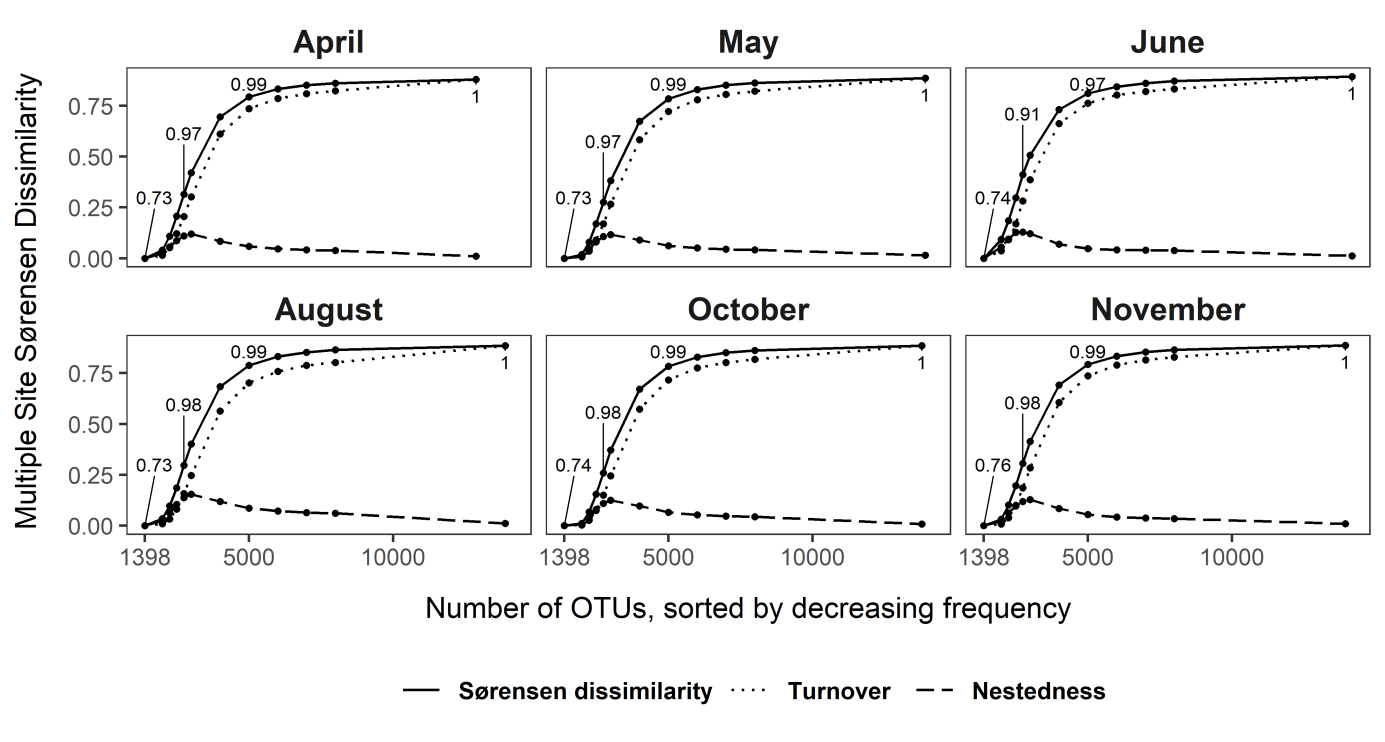


**Supplementary Figure 5. Abundance-independent β-diversity, expressed as multiple site dissimilarities using the Sørensen-Index, partitioned into components of turnover and nestedness (Baselga, 2010). The indices were calculated for subsets containing increasing number of OTUs, which were before sorted by decreasing site frequency. 1398 OTUs were present in all samples and composed a subset of resident OTUs, for which the species turnover must be zero (β_Sør_ = 0). Labels inside the plot indicate the accumulated abundance-unweighted relative abundance, which was represented by each subset. "1" marks the result of the total community.**


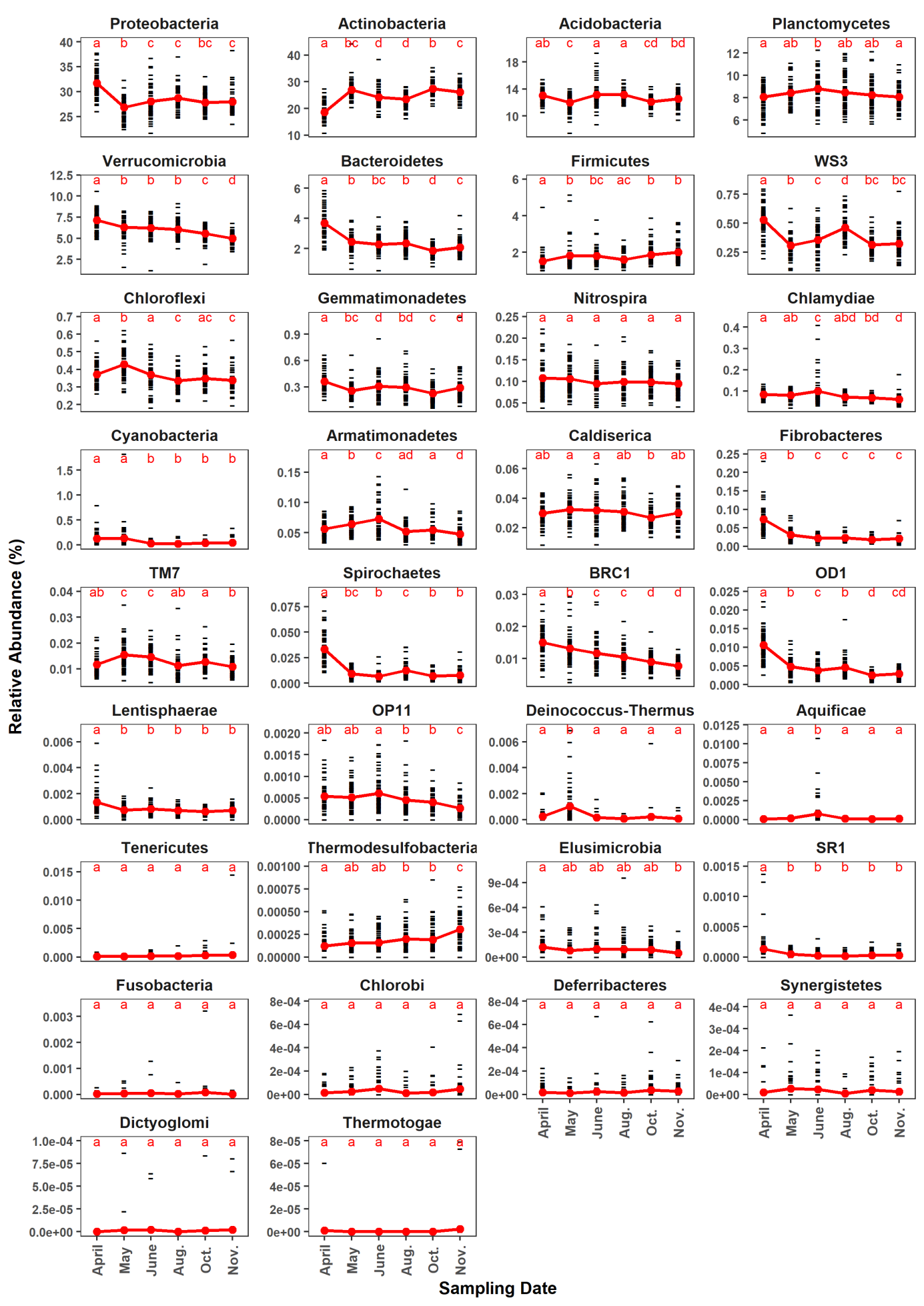


Supplementary Figure 6. Changes in relative abundances on phylum level. Annotated by RDP-Classifier on all reads (before OTU clustering). Count data scaled to all bacterial observations. Red lines indicate the mean at each sampling date. Shared letters represent groups of statistically insignificant pairwise comparisons (contrasts of estimated marginal means on generalized least squares models after correction for spatial autocorrelation).


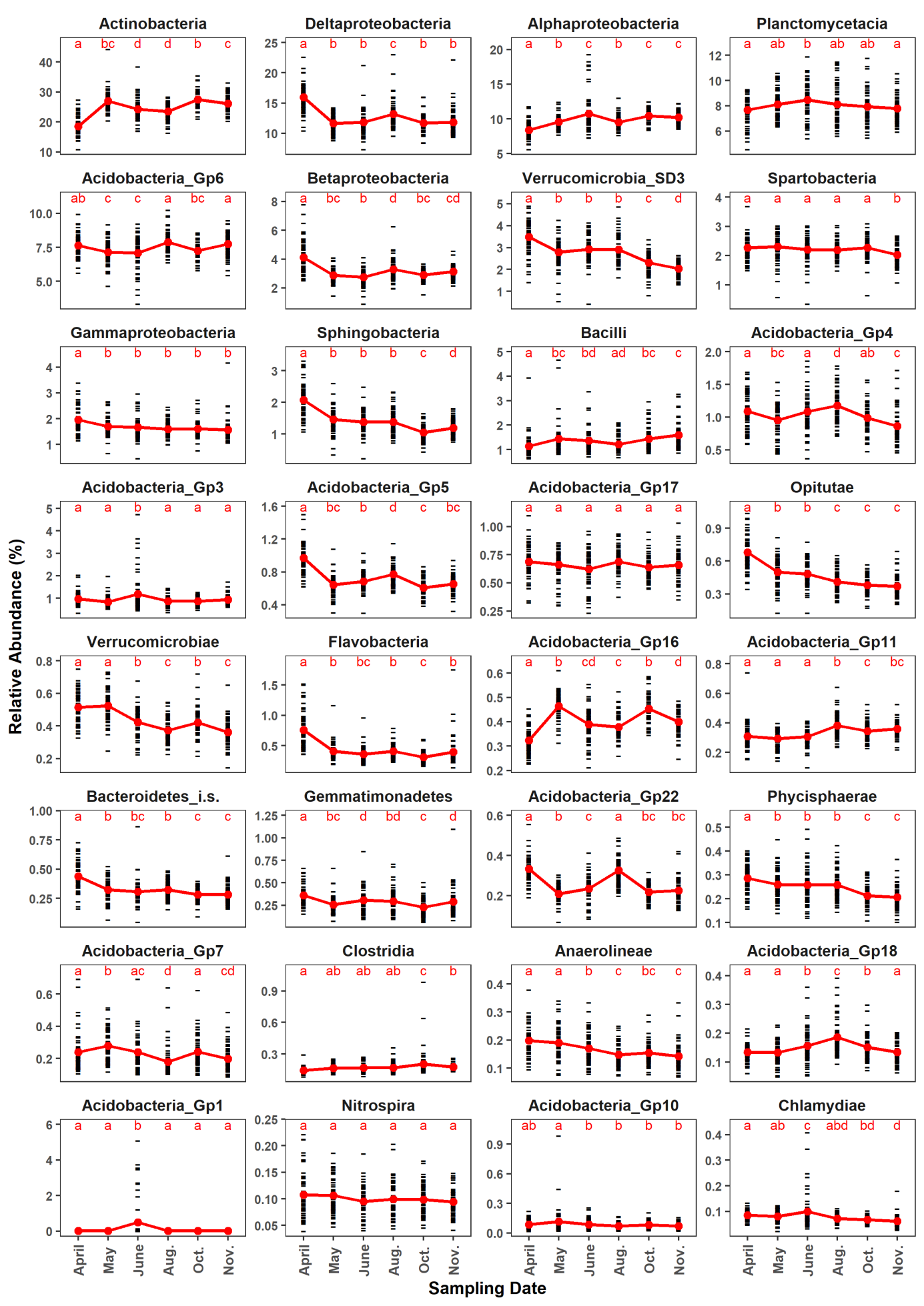


**Supplementary Figure 7. Changes in relative abundances for the 32 most abundant sub-phylum groups (mostly class level) across the sampling season, as annotated by RDP-Classifier on all reads (before OTU clustering). Count data scaled to all bacterial observations. Red lines indicate the mean at each sampling date. Shared letters represent groups of statistically insignificant pairwise comparisons (contrasts of estimated marginal means on generalized least squares models after correction for spatial autocorrelation).**


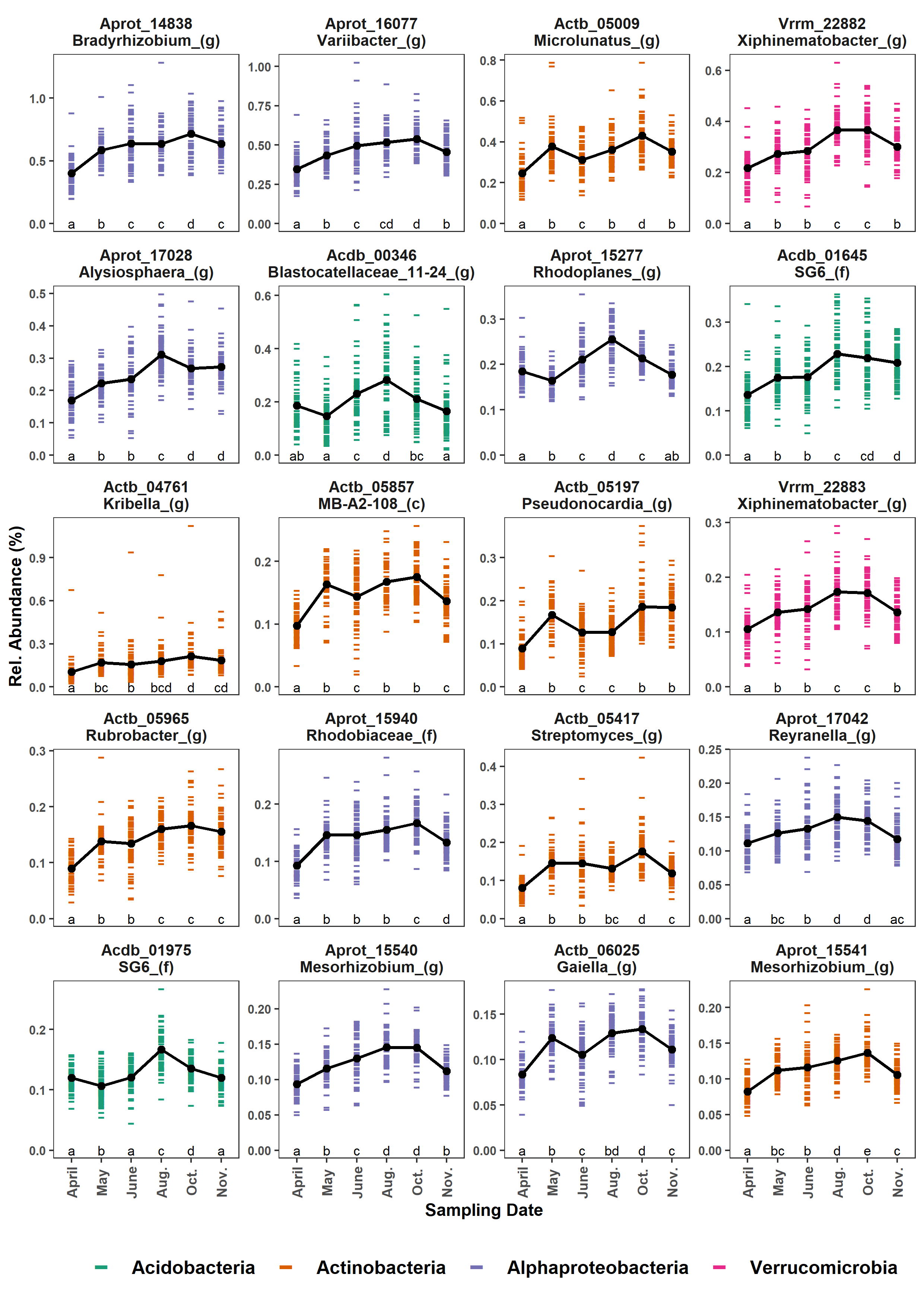


Supplementary Figure 8. Relative abundances of the 20 most abundant OTUs (total fraction of all bacterial reads > 0.1%, in all cases) across the sampling seasons. Annotation to the last known rank ((g) = genus, (f) = family). Count data scaled to all bacterial observations. Black lines follow the mean at each sampling date. Shared letters represent groups of statistically insignificant pairwise comparisons (contrasts of estimated marginal means on generalized least squares models after correction for spatial autocorrelation).


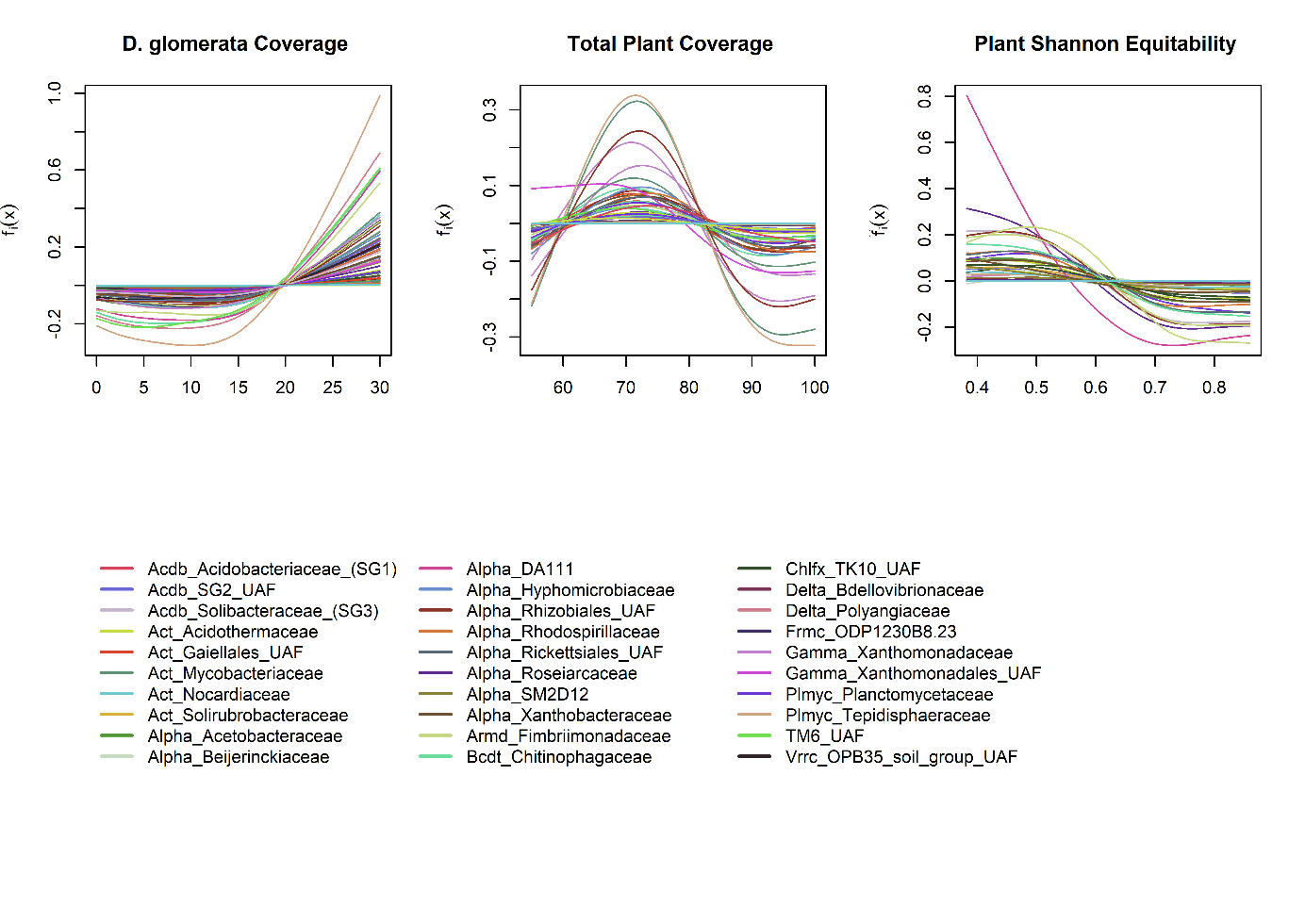


Supplementary Figure 9. Partial additive functions of OTUs, which were differentially active in irregular sites and their environment interactions. In each panel, the partial response functions of OTUs to a given environmental parameter are overlaid. The y-coordinate should be interpreted as the effect of the partial function after adjusting for all other variables (on log(y), with y being the absolute read count of the OTU, scaled to total bacterial read counts), centered around zero. Note the individual scales of each panel. UAF = Unassigned family within the last known taxon.


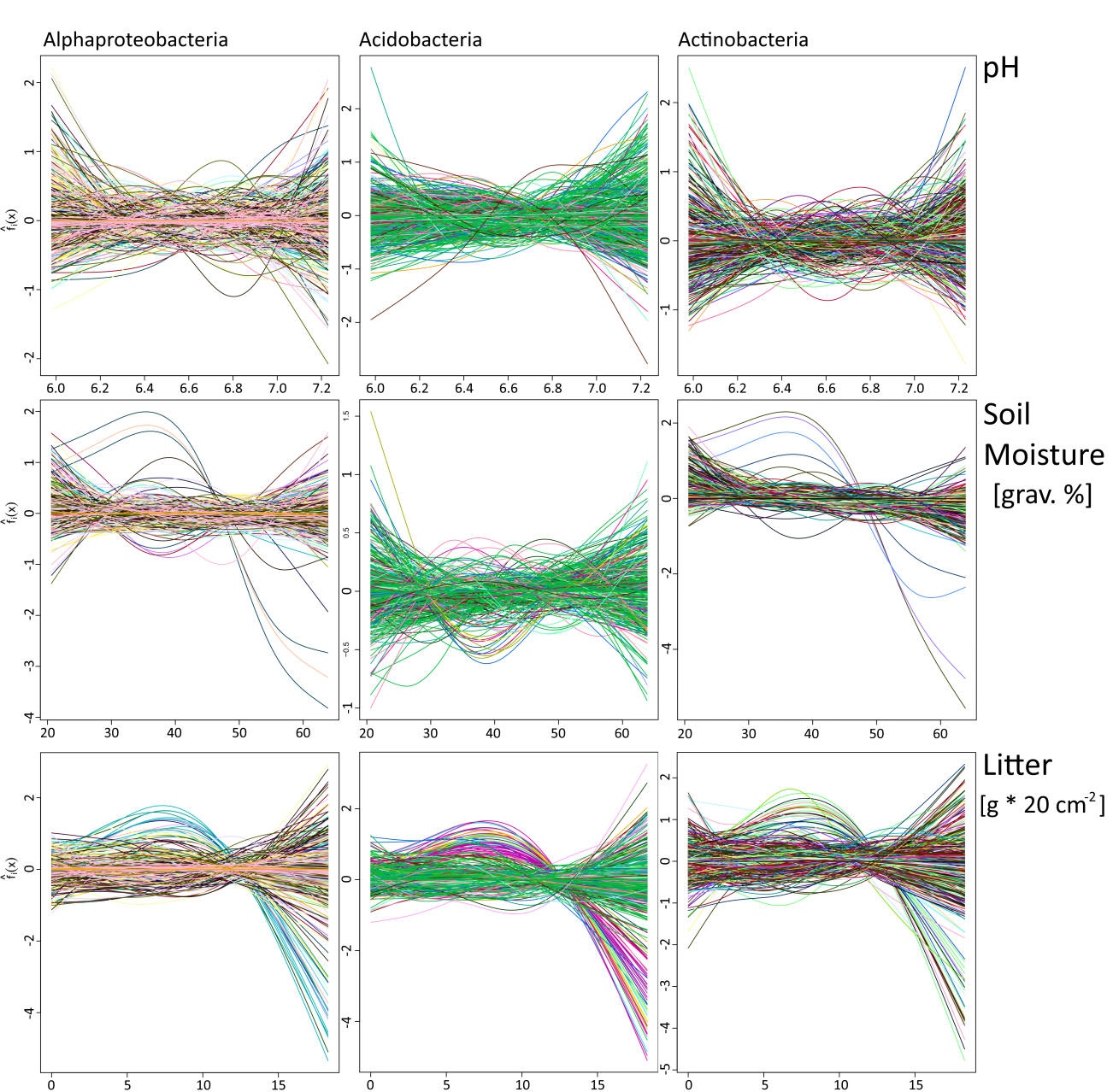

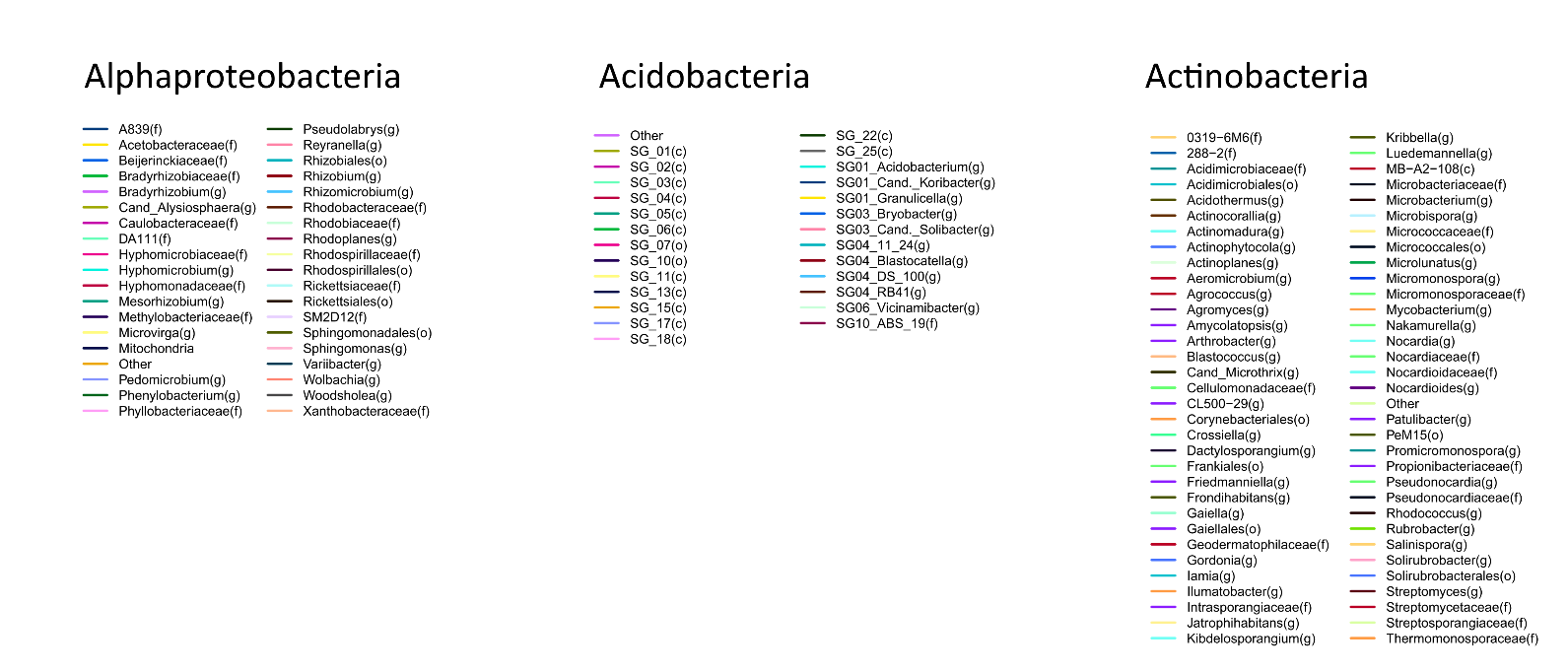


**Supplementary Figure 10. Partial additive functions of selected bacteria – environment interactions. In each panel, the partial response functions of single OTUs to a given environmental parameter are overlaid. The y-coordinate should be interpreted as the effect of the partial function after adjusting for all other variables (on log(y), with y being the absolute read count of the OTU, scaled to total bacterial read counts), centered around zero.**

# **References**:

Benjamini, Y., Hochberg, Y., 1995. Controlling the False Discovery Rate: A Practical and Powerful Approach to Multiple Testing. *Journal of the Royal Statistical Society. Series B ((Statistical Methodology)* 57, 289-300.

Chantigny, M.H., 2003. Dissolved and water-extractable organic matter in soils: a review on the influence of land use and management practices. *Geoderma* 113, 357-380.

Chao, A., Gotelli, N.J., Hsieh, T.C., Sander, E.L., Ma, K.H., Colwell, R.K., Ellison, A.M., 2014. Rarefaction and extrapolation with Hill numbers: a framework for sampling and estimation in species diversity studies. *Ecological Monographs* 84, 45-67.

Eilers, P.H.C., Marx, B.D., 1996. Flexible smoothing with B-splines and penalties. *Statistical Science* 11, 89-102.

Gotelli, N.J., Graves, G.R., 1996. Null Models in Ecology. Smithsonian Institution Press, Washington, D.C., USA.

Herberich, E., Sikorski, J., Hothorn, T., 2010. A robust procedure for comparing multiple means under heteroscedasticity in unbalanced designs. *PLOS ONE* 5, e9788.

Hofner, B., Boccuto, L., Goker, M., 2015. Controlling false discoveries in high-dimensional situations: boosting with stability selection. *BMC Bioinformatics* 16, 144.

Hofner, B., Mayr, A., Robinzonov, N., Schmid, M., 2014. Model-based boosting in R: a hands-on tutorial using the R package mboost. *Computational Statistics* 29, 3-35.

Hothorn, T., Bretz, F., Westfall, P., 2008. Simultaneous inference in general parametric models. *Biometric Journal* 50, 346-363.

Kneib, T., Hothorn, T., Tutz, G., 2009. Variable Selection and Model Choice in Geoadditive Regression Models. *Biometrics* 65, 626-634.

Lenth, R., 2018. emmeans: Estimated Marginal Means, aka Least-Squares Means. R-package

Love, M.I., Huber, W., Anders, S., 2014. Moderated estimation of fold change and dispersion for RNA-seq data with DESeq2. *Genome Biology* 15.

Mandal, S., Van Treuren, W., White, R.A., Eggesbo, M., Knight, R., Peddada, S.D., 2015. Analysis of composition of microbiomes: a novel method for studying microbial composition. *Microbial Ecology in Health and Disease* 26, 27663-.

Mayr, A., Binder, H., Gefeller, O., Schmid, M., 2014. The evolution of boosting algorithms. From machine learning to statistical modelling. *Methods of Information in Medicine* 53, 419-427.

Mayr, A., Hofner, B., Schmid, M., 2012. The Importance of Knowing When to Stop. A Sequential Stopping Rule for Component-wise Gradient Boosting. *Methods of Information in Medicine* 51, 178-186.

Meinshausen, N., Bühlmann, P., 2010. Stability selection. *Journal of the Royal Statistical Society: Series B (Statistical Methodology)* 72, 417-473.

Parks, D.H., Tyson, G.W., Hugenholtz, P., Beiko, R.G., 2014. STAMP: statistical analysis of taxonomic and functional profiles. *Bioinformatics* 30, 3123-3124.

Regan, K.M., Nunan, N., Boeddinghaus, R.S., Baumgartner, V., Berner, D., Boch, S., Oelmann, Y., Overmann, J., Prati, D., Schloter, M., Schmitt, B., Sorkau, E., Steffens, M., Kandeler, E., Marhan, S., 2014. Seasonal controls on grassland microbial biogeography: Are they governed by plants, abiotic properties or both? *Soil Biology and Biochemistry* 71, 21-30.

Robinson, M.D., McCarthy, D.J., Smyth, G.K., 2010. edgeR: a Bioconductor package for differential expression analysis of digital gene expression data. Bioinformatics 26, 139-140.

Schmid, M., Hothorn, T., 2008. Boosting additive models using component-wise P-Splines. *Computational Statistics & Data Analysis* 53, 298-311.

Shah, R.D., Samworth, R.J., 2013. Variable selection with error control: another look at stability selection*. Journal of the Royal Statistical Society: Series B (Statistical Methodology)*75, 55-80.

Stegen, J.C., Lin, X., Fredrickson, J.K., Chen, X., Kennedy, D.W., Murray, C.J., Rockhold, M.L., Konopka, A., 2013. Quantifying community assembly processes and identifying features that impose them. *The ISME Journal* 7, 2069-2079.

Stegen, J.C., Lin, X., Fredrickson, J.K., Konopka, A., 2015. Estimating and Mapping Ecological Processes Influencing Microbial Community Assembly. *Frontiers in Microbiology* 6.

Tucker, C.M., Shoemaker, L.G., Davies, K.F., Nemergut, D.R., Melbourne, B.A., 2016. Differentiating between niche and neutral assembly in metacommunities using null models of β-diversity. *Oikos* 125, 778-789.

Zhou, J., Ning, D., 2017. Stochastic Community Assembly: Does It Matter in Microbial Ecology? *Microbiology and Molecular Biology Reviews* 81.
